# Positive-unlabeled learning for disease gene identification

**DOI:** 10.1093/bioinformatics/bts504

**Published:** 2012-08-24

**Authors:** Peng Yang, Xiao-Li Li, Jian-Ping Mei, Chee-Keong Kwoh, See-Kiong Ng

**Affiliations:** ^1^Bioinformatics Research Centre, School of Computer Engineering, Nanyang Technological University, Singapore, 639798 and ^2^Institute for Infocomm Research, A*Star, Singapore, 138632, Singapore

## Abstract

**Background:** Identifying disease genes from human genome is an important but challenging task in biomedical research. Machine learning methods can be applied to discover new disease genes based on the known ones. Existing machine learning methods typically use the known disease genes as the positive training set *P* and the unknown genes as the negative training set *N* (non-disease gene set does not exist) to build classifiers to identify new disease genes from the unknown genes. However, such kind of classifiers is actually built from a noisy negative set *N* as there can be unknown disease genes in *N* itself. As a result, the classifiers do not perform as well as they could be.

**Result:** Instead of treating the unknown genes as negative examples in *N*, we treat them as an unlabeled set *U*. We design a novel positive-unlabeled (PU) learning algorithm PUDI (PU learning for disease gene identification) to build a classifier using *P* and *U*. We first partition *U* into four sets, namely, reliable negative set *RN*, likely positive set *LP*, likely negative set *LN* and weak negative set *WN*. The weighted support vector machines are then used to build a multi-level classifier based on the four training sets and positive training set *P* to identify disease genes. Our experimental results demonstrate that our proposed PUDI algorithm outperformed the existing methods significantly.

**Conclusion:** The proposed PUDI algorithm is able to identify disease genes more accurately by treating the unknown data more appropriately as unlabeled set *U* instead of negative set *N*. Given that many machine learning problems in biomedical research do involve positive and unlabeled data instead of negative data, it is possible that the machine learning methods for these problems can be further improved by adopting PU learning methods, as we have done here for disease gene identification.

**Availability and implementation:** The executable program and data are available at http://www1.i2r.a-star.edu.sg/∼xlli/PUDI/PUDI.html.

**Contact:** xlli@i2r.a-star.edu.sg or yang0293@e.ntu.edu.sg

**Supplementary information:** Supplementary Data are available at *Bioinformatics* online.

## 1 INTRODUCTION

Uncovering the causative genes for human diseases has significant impact to healthcare since many medical conditions are in some way influenced by human genetic variations. In recent years, an increasing number of genes have been confirmed as causative genes to diseases. This provides an invaluable resource for developing machine learning methods to identify novel disease genes from the vast number of unknown genes in the genome, using the confirmed disease genes as positive training examples.

Recent studies have revealed that genes associated with similar disorders have been shown to demonstrate higher probabilities of similar gene expression profiling (Ala *et al.*, 2008), high functional similarities (Ideker and Sharan, 2008) and physical interactions between their gene products (Brunner *et al.*, 2004; Goh *et al.*, 2007). As such, those unknown genes that share similar gene expression profiles with the confirmed disease genes, have high functional similarities with disease genes and interact with disease gene products are likely to be disease genes as well. Ala *et al.* (2008) systematically integrated human–mouse conserved similar expression profiles with phenotype similarity map to rank potential disease genes in large genomic regions. Köhler *et al.* (2008) made use of the observation that proteins caused by same/similar disorders are likely attached together in protein–protein interaction (PPI) network (Gandhi *et al.*, 2006) and applied the random walk algorithm on the PPI network for disease gene prioritization. More recently, Yang *et al.* (2011) proposed a network propagation-based method RWPCN on a novel protein complex network for prioritizing disease genes. In the above two PPI network-based approaches, those unknown genes directly interact with one or multiple confirmed disease genes are likely to be predicted as candidate disease genes.

Note that the above methods only provide a gene rank list and a threshold is needed to decide whether a specific gene is disease related or not. A more biologically meaningful approach would be to build a binary classification model that can automatically classify a gene as disease or not. This requires identifying systematic differences between disease genes (positive class) and non-disease genes (negative class). López Bigas and Ouzounis (2004) investigated the distinguishing features of protein sequences between disease and non-disease genes and found that compared to the products of non-disease genes, proteins involved in hereditary diseases tend to be long, with more homologs with distant species, but fewer paralogs within human genome. Adie *et al.* (2005) further improved on this method by employing a decision tree algorithm based on a variety of genomic and evolutionary features, such as coding sequence length, evolutionary conservation, presence, closeness of paralogs in the human genome, etc. In addition to sequence information, proteins’ topological information in protein interaction networks has also been shown to be useful for evaluating the likelihood that an unknown gene is disease related or not. In particular, Xu *et al.* (2006) employed the K-nearest neighbor (KNN) classifier to predict disease genes based on the topological features in PPI networks, such as proteins’ degree, the percentage of disease genes in proteins’ neighborhood, etc. Smalter *et al.* (2007) applied support vector machines (SVMs) classifier using PPI topological features, sequence-derived features, evolutionary age features, etc. Radivojac *et al.* (2008) first built three individual SVM classifiers using three types of features, i.e. PPI network, protein sequence and protein functional information, respectively. It then built a final classifier by combining the predictions from three individual classifiers for candidate gene prediction.

The above works employed machine learning methods to build a binary classifier by using the confirmed disease genes as positive training set *P* and some unknown genes as negative training set *N*. However, since the negative set *N* will contain unconfirmed disease genes (false negatives), which confuses the machine learning techniques for building accurate classifiers. As such, the classifiers built based on the positive set *P* and noisy negative set *N* do not perform as well as they could in identifying new disease genes.

Recently, Mordelet *et al.* proposed a bagging method ProDiGe for disease gene prediction. This method iteratively chooses random subsets (*RS*) from *U* and trains multiple classifiers using bias SVM to discriminate *P* from each subset *RS*. It then aggregates all the classifiers to generate the final classifier (Mordelet *et al.*, 2011). However, as the random subsets *RS* from *U* could still contain unknown disease genes, individual classifiers are thus not accurate and this will affect the overall performance of the final classifier. In addition, ProDiGe method treats all the examples in *RS*/*U* homogeneously. Since we can compute the similarities between the examples in *U* and the positive examples in *P*, we can thus estimate the probabilities of the examples in *U* belonging to positive/negative class. As such, the examples in *U* can be partitioned into different subsets and subsequently be treated heterogeneously for classifier building.

In this article, we design a novel positive-unlabeled (PU) learning algorithm PUDI (PU learning for disease gene identification) to build a more accurate classifier based on *P* and *U* (Li *et al.*, 2003, 2007, 2009). First, we use a comprehensive combination of biological process, molecular function, cellular component, protein domain and PPI data to represent the genes into feature vectors. We design a novel feature selection method to reduce the dimensionality of the feature vectors. Then, we partition *U* into four label sets, namely, reliable negative set, likely positive set, likely negative set, and weak negative set, based on their likelihoods being positive/negative class. Finally, we build multi-level weighted SVMs using these four sets together with positive set *P* for identifying disease genes.

To the best of our knowledge, PUDI is the first to design a novel multi-level PU learning algorithm for building a classifier for disease gene identification. We have compared PUDI with three state-of-the-art techniques, namely, Smalter’s method (Smalter *et al.*, 2007), Xu’s method (Xu *et al.*, 2006) and ProDiGe method. Our experimental results showed that PUDI outperforms the existing methods significantly for predicting *general* disease genes and for identifying disease genes in eight *specific* disease classes, such as cardiovascular diseases, endocrine diseases, psychiatric diseases, metabolic diseases and cancer, etc.

## 2 METHODS

In Section 2.1, we introduce a method to characterize genes into feature vectors using different biological features. In Section 2.2, we propose a novel feature selection method to choose distinguishing features for better classification. Finally, we describe our proposed positive unlabeled learning procedure in Section 2.3. The system schema and data flow of PUDI are shown in Supplementary Figures S2 and S3, respectively.

### 2.1 Gene characterization

Our approach is to characterize genes (or corresponding gene products) using a comprehensive range of biological information. The information include protein domains (*D*), molecular functions (*MF*), biological processes (*BP*), cellular components (*CC*), as well as the genes’ corresponding topological properties in the protein interaction networks (*PPI*). In other words, each gene *g_i_* is represented as a vector *Vg_i_* which consists of a *domain* component *Dg_i_*, a *molecular function* component *MFg_i_*, a *biological process* component *BPg_i_*, a *cellular component* component *CCg_i_* and a *protein interaction* component *PPIg_i_*, i.e. *Vg_i_* = (*Dg_i_*, *MFg_i_*, *BPg_i_*, *CCg_i_*, *PPIg_i_*). We describe each of these components in details below.

Protein domains are evolutionarily conserved modules of amino acid sub-sequence postulated that as nature’s functional ‘building blocks’ for constructing the vast array of different proteins. Protein domains are thus regarded as essential units for such biological functions as the participation in transcriptional activities and other intermolecular interactions. Databases, such as the protein families (Pfam) database and others, have been compiled to comprise comprehensive information about domains (http://www.sanger.ac.uk/Software/Pfam) (Finn *et al.*, 2010). In this study, we only used Pfam-A, a collection of manually curated and functionally assigned domains, instead of Pfam-B, which is computationally derived collection of domains (and hence less accurate), to ensure accuracy in our predictions. The *domain* component *Dg_i_* of the given gene *g_i_* is represented as *Dg_i_* = (*d_i_*_1_, *d_i_*_2_, … , *d_i_*_|Pfam-A|_) where *d_ij_* (1 ≤ *j ≤ *|Pfam-A|) is equal to 1 if *g_i_*s gene product contains the corresponding domain in Pfam-A; 0 otherwise.

For the *molecular function* component *MFg_i_*, *biological process* component *BPg_i_* and *cellular component CCg_i_*, we use the Gene Ontology (GO, http://www.geneontology.org/) database, which provides a common vocabulary that can be used to describe the biological processes (*BP*), molecular functions (*MF*) and cellular components (*CC*) for the genes (Harris *et al.*, 2004).

Let *SMF = *{*MF*_1_, *MF*_2_, … , *MF*_|_*_SMF_*_|_}, *SBP =*{*BP*_1_, *BP*_2_, … , *BP*_|_*_SBP_*_|_)} and *SCC* =**{*CC*_1_, *CC*_2_, … , *CC*_|_*_SCC_*_|_} represent the set of *MF*, *BP* and *CC* in GO, respectively. Then *MFg_i_* = (*mf_i_*_1_, *mf_i_*_2_, … , *mf_i_*_|_*_SMF_*_|_), *BPg_i_* = (*bp_i_*_1_, *bp_i_*_2_, … , *bp_i_*_|_*_SBP_*_|_), *CCg_i_* = (*cc_i_*_1_, *cc_i_*_2_, … , *cc_i_*_|_*_SCC_*_|_). Let us take *MFg_i_* as an example (similar for *BPg_i_*, *CCg_i_*) to show how to compute each element *mf_ij_* (1 ≤ *j ≤ *|*SMF*|). Note that each *g_i_* can be annotated by many GO terms at different levels in GO's DAG structure (Direct Acyclic Graphs). For example, the gene ADH4 is annotated by molecular function term set {0004022, 004024, 0004174, 0046872, 0008270, 0004023} in the GO database. Assume that *g_i_* has the following molecular functions *FUNg_i_* = {*fun*_1_, *fun*_2_, … , *fun_k_*}, *mf_ij_* can be computed as follows:
(1)
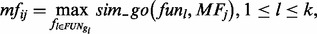

where *sim_go*( *fun_l_*, *MF_j_*) is the GO term similarity between two functions *fun_l_* and *MF_j_*. Since the GO terms of BP, MF and CC are organized into DAG structure, we use the computational method proposed in (Wang *et al.*, 2007) to compute the similarity between two GO terms *A* and *B*. Let the GO term *A* be represented as 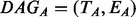, where *T_A_* includes term *A* and all of its ancestor GO terms in the DAG graph and *E_A_* is the set of edges (semantic relations) connecting the GO terms in *T_A_*. For a term *t* in 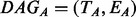, its *S*-value related to term A, *S_A_*(*t*), is defined as:
(2)
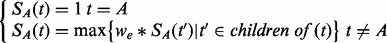

where *w_e_* is the weight for edge 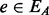 linking term *t* with its child term *t*′. The weights *w_e_* for two types of edges ‘*is a*’ and ‘*part of*’ are assigned as 0.8 and 0.6, respectively, as recommend in (Wang *et al.*, 2007).

Given 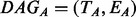 and 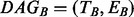 for GO terms *A* and *B*, respectively, the similarity between *A* and *B*, *sim* (*A*, *B*), is defined as:
(3)
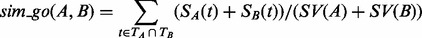

where 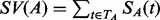.

For the *protein interaction* component *PPIg_i_*, we exploit a protein interaction network *G_PPI_* = (*V_PPI_ E_PPI_*) where *V_PPI_* represents the set of the interacting proteins and *E_PPI_* denotes all the detected pairwise interactions between proteins in *V_PPI_*. We use four topological features from *G_PPI_* (Xu *et al.*, 2006) for gene *g_i_* as *PPIg_i_* = (*degree_i_*, *1N_i_*, *2N_i_*, *Cluster_i_*).

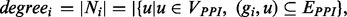

where *N_i_* is the set of *g_i_*'s direct neighbors in *G_PPI_* and degree of *g_i_* is the cardinality of *N_i_*. *1N_i_* represents the proportion of disease genes in *N_i_* which is defined as 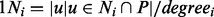. Similarly, *2N_i_* represents the proportion of disease genes in *g_i_*'s larger neighborhood (with radius 2, i.e. including *g_i_*'s direct neighbors and indirect neighbors). *Cluster_i_* is the clustering coefficient which measures the degree to which *g_i_*'s direct neighbors in *G_PPI_* tend to cluster together (Watts and Strogatz, 1998).

### 2.2 Feature selection

We have represented each gene *g_i_* using a comprehensive list of biological features. Supplementary Table S1 lists the numbers of features for each category, showing large numbers of features for *BP*, *MF*, *CC* and domain *D* (For PPI, we only have four features). In this section, we propose a novel feature selection method to choose subsets of features that are useful for distinguishing disease genes from non-disease genes.

For each feature *f* in *BP*, *MF*, *CC* and *D*, we compute its *affinity frequency* in the positive set *P af* ( *f*, *P*) and the unlabeled set *U af* ( *f*, *U*):
(4)
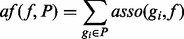

(5)
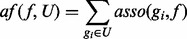

where 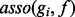 is the association score between a gene *g_i_* in *P* (or *U*) and the feature *f*. If ( *f* ∈ *BP* U *MF* U *CC*), then
(6)
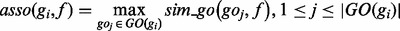



In other words, we compute the association score using the maximal GO term similarity between feature *f* and each of the *g_i_*'s GO terms.

In the case of 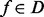, 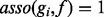 if 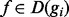 (or feature *f* belongs to gene *g_i_*'s domain set); 0 otherwise.

We evaluate each feature *f* by its *discrimination ability score*:
(7)
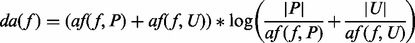



Our objective is to choose those *distinguishing features* that either *frequently occurred* in the disease gene set *P* but seldom occurred in unlabeled gene set *U* (assuming large portion of unknown genes are still negatives) or frequently occurred in *U* but seldom occurred in *P*. In this way, we choose the features which can help us to distinguish disease genes from non-disease genes. Let us see how Equation (7) helps us do that. We can see from the equation that given a feature *f*, if its *affinity frequency* in *P af*( *f*, *P*) is large while its frequency in *U af* ( *f*, *U*) is small or the frequency in *U af*( *f*, *U*) is large while the frequency in *P af* ( *f*, *P*) is small, then the value of *da*( *f* ) will be large since both factors 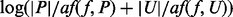 and 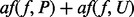 are large. When *af*( *f*, *P*) and *af*( *f*, *U*) are both large, then the value of 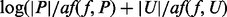 will be small, hence, *da*( *f* ) will be relatively small. Similarly, when *af*( *f*, *P*) and *af*( *f*, *U*) are both small, the value of 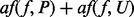 will be small and *da*( *f* ) will also be relatively small.

With a reduced feature set formed by Equation (7), we are able to speed up the computation for building a classification model, as well as avoid potential model over-fitting. Supplementary Tables S2 and S3 list some examples of highly ranked GO and domain features, indicating the features selected are indeed associated with various diseases.

### 2.3 PU learning to identify the disease genes from U

With the above feature representation and feature selection methods, we are now ready to build a classifier using the given confirmed disease gene set *P* and unlabeled gene set *U*. We call our proposed algorithm PUDI. Given that we do not have any negative genes, the first step is to extract a set of *reliable negative* genes *RN* from *U* by computing the similarities of the unlabeled genes in *U* with the positive genes in *P*, based on the idea that those genes in *U* that are very dissimilar to the genes in *P* are likely to be reliable negatives (Li *et al.*, 2003).

**Fig. 1.**
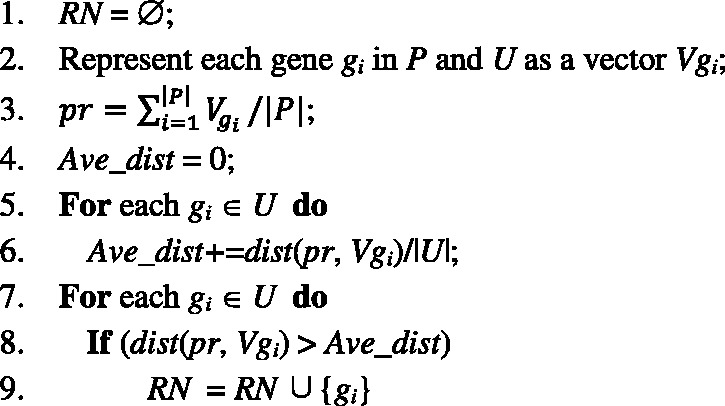
Extract reliable negative gene set (*RN*) from U

The detailed algorithm is given in Figure 1. We initialize the reliable negative set *RN* as an empty set and represent each gene *g_i_* in *P* and *U* as a vector *Vg_i_* using the feature representation method discussed in Section 2.1 and the feature selection method presented in Section 2.2. We build a ‘positive representative vector’ (*pr*) by summing up the genes in *P* and normalizing it (Line 3). Lines 4–6 compute the average distance of each gene *g_i_* in *U* from *pr* using the *Euclidean distance*, *dist*(*pr*, *Vg_i_*) (Deza and Deza, 2009). For each gene *g_i_* in *U*, if its Euclidean distance *dist*(*pr*, *Vg_i_*) > *Ave*_*dist*, we regard it as a *reliable* negative example and store it in *RN* (Lines 7–9); since it is very far away from the positive examples, it is thus safe for us to treat it as a negative example.

At this point, we have a positive set *P*, a reliable negative set *RN* and a refined unlabeled set *U-RN*, so we can build a classifier using *P* and *RN* with any supervised learning method. However, the reliable negatives in *RN* may still be far away from the desired boundary between the actual positive and negative data. To build a robust classifier, an important next step in our PUDI algorithm is to further extract the likely positive examples *LP* and the likely negative examples *LN* from genes in the *U-RN* which are near the positive and negative classification boundary.

To do so, we construct a gene similarity network 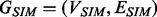, in which a vertex *v* in vertex set *V_SIM_* represents a gene in *P* U *U* and an edge (*g_i_*, *g_j_*) in edge set *E_SIM_* represents a connection between two distinct genes *g_i_* and *g_j_*. To construct *G_SIM_*, we define the pairwise similarity matrix W_*ij*_ between any two genes *g_i_* and *g_j_* as follows:
(8)
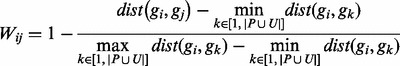



A high value in *W_ij_* indicates that the two genes *g_i_* and *g_j_* share the similar biological evidence and thus likely belong to same category (disease or non-disease). For each gene 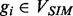, we connect it with another gene if their similarities are among top *Q* most similar ones to gene *g_i_*. This is to ensure that we keep only those robust connections in the network. With the resulting gene similarity network 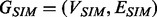, we can then perform a random walk with restart algorithm to detect the likely positives and likely negatives, as follows:

*Step 1. Initialize the prior probabilities of positives and reliable negatives.* Let *P*_0_ and *N*_0_ denote the prior probability vector of the positives and reliable negatives, respectively. In *P*_0_ the prior probabilities of positive examples in *P* are assigned an equal probability + 1 (with the sum of the probabilities equal to |*P*|). In *N*_0_, the prior probabilities of the reliable negative examples in *RN* are assigned as −|*P*|/|*RN*| (so the sum of the probabilities equals to −|*P*|). This guarantees fair allocation of prior probabilities from the two sets of labeled data. We represent the overall prior probability vector for the training data as 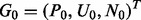, where 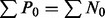. The prior probabilities in *U*_0_ are assigned 0 and we will decide their posterior probabilities in Step 2.

*Step 2. Propagate the label information influence from G_0_ to the genes of U-RN in the network*. After initialing the prior probabilities for positive examples and reliable negative examples as above, we score all the remaining unlabeled genes in the network by propagation. We propose to do flow propagation for this and adopt the Random Network algorithm (Lovász, 1993) to our network *G_SIM_*. The prior influence flows of labeled genes are distributed to their neighbors, which continue to spread the influence flows to other nodes iteratively. Formally, let *G*_0_ be the initial probability vector, *G_r_*, the probability vector at step *r*, can be calculated as follows:
(9)
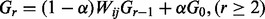

where 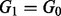 and 
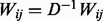
. Here *D* is the diagonal matrix with 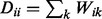. The parameter *α* provides a probabilistic weighting of the prior information returning back to initial genes at every step. In this work, we set parameter *α* to 0.8, as recommend in (Li and Patra, 2010). At the end of the iterations, the prior information held by every vertex/gene in the network will reach a steady state as proven by (Lovász, 1993). This is determined by the probability difference between *G_r_* and 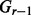, represented as 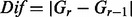 (measured by L1 norm). When 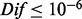 (Köhler *et al.*, 2008), we consider that a steady stage has been reached and terminated the iterative process.

*Step 3. Label the likely positives and likely negatives.* According to the posterior probabilities of *U*_0_, we further partition the remaining unlabeled data *U-RN* data set into three parts: likely positives (*LP*), likely negative (*LN*) and weak negative (*WN*) using the following criteria:
(10)
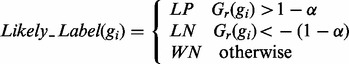



We can now build a classifier using the given positive set *P* and four extracted sets from *U*, namely, the reliable negative set *RN*, the likely positive set *LP*, the likely negative set *LN* and the weak negative set *WN*. To take into account of the inherently different levels of trustworthiness of labels in *P*, *RN*, *LP*, *LN* and *WN*, we use a multi-level examples learning technique, weighted SVMs (Chang and Lin, 2011; Vapink, 1998), to build a classifier. The objective function of weighted SVM can be defined as (Liu *et al.*, 2011):
(11)
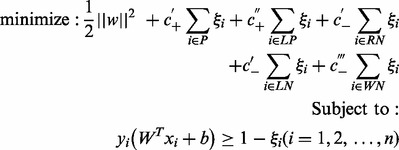

where *ξ_i_* is a slack variable which allows the misclassification of some training examples, and 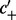, 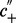, 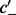, 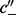 and 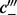 represent the penalty factors for SVM to penalize the wrongly classified examples in *P*, *LP*, *RN*, *LN* and *WN*, respectively. In particular, 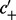 > 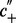
since we are more confident with positive set *P* than the likely positive set *LP.* Correspondingly, we give a larger penalty if examples from *P* are classified as negative class than if examples from *LP* are classified as negative class. Similarly, condition 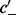 > 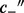 > 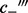 holds since we are more confident with *RN* than *LN* and we are also more confident with *LN* than *WN*. We used 10-fold cross validation to decide the values for these penalty factors—please refer to Section 3 in our Supplementary Material for details.

## 3 RESULTS

In this section, we present our experimental results on the comparisons of our proposed PUDI method with state-of-the-art techniques on *general* disease genes prediction, feature selection, parameter sensitivity analysis, *specific* disease gene prediction and *novel* disease gene prediction.

### 3.1 Experimental data, settings and evaluation metrics

#### 3.1.1 Experimental data

We downloaded the latest versions of disease gene data from GENECARD (Safran *et al.*, 2010) and OMIM (McKusick, 2007). GENECARD and OMIM were then combined into our disease gene benchmark. There are 5405 known disease genes spanning 2751 disease phenotypes after combining GENECARD data together with OMIM. Gene Ontology, consisting of three sub-ontology *MF*, *BP* and *CC* are downloaded from GO (http://www.geneontology.org/). Protein domains were obtained from http://www.sanger.ac.uk/Software/Pfam (Finn *et al.*, 2010). Human PPI data were downloaded from the HPRD (Prasad *et al.*, 2009) and OPHID (Brown *et al.*, 2005). The combined PPI data set contained 143 939 PPIs involving a total of 13 035 human proteins.

#### 3.1.2 Experimental settings

We chose the known disease genes with at least two-thirds non-zero features as our positive training set *P.* Here, |*P*|**=**3849 since not all the genes possess the *MF*, *BP*, *CC*, *D* and PPI features in the current data sources. We used ∼16 k genes from Ensembl (Flicek *et al.*, 2011) as the unknown gene set from which we randomly select the actual unlabeled set so that we have a balanced |*P*|**=**|*U*|, following the setting in (Adie *et al.*, 2005; Smalter *et al.*, 2007; Xu *et al.*, 2006).

We then performed feature selection and selected the top *N* scored features (the default value of *N* is 1000) for each of the four feature groups, i.e. *BP*, *MF*, *CC* and *D*, respectively. We executed 10-fold cross validation experiments to evaluate the performance of all the techniques on predicting general disease genes, and 3-fold cross validation on predicting disease genes for specific disease groups. The average results are reported in Section 3.2.

#### 3.1.3 Evaluation metrics

We use the F-measure (Bollmann *et al.*, 1981) to evaluate the performance of our classification systems. The F-measure is the harmonic mean of precision (*p*) and recall (*r*) and it is defined as *F*=**2**× *p*× *r*/(*p*+ *r*). The F-measure reflects an average effect of both precision and recall. When either of them (*p* or *r*) is small, the value will be small. Only when both of them are large, the F-measure will be large. This is suitable since having either too small a precision or too small a recall for disease gene prediction is unacceptable and would be reflected by a low F-measure.

### 3.2 Experimental results

First, we compared our proposed PUDI algorithm with three state-of-the-art techniques, namely, Smalter’s method (Smalter *et al.*, 2007), Xu’s method (Xu *et al.*, 2006) and ProDiGe method (Mordelet *et al.*, 2011) for predicting *general disease genes*, i.e. automatically classify an unknown gene into a disease gene or a non-disease gene. We employed 10-fold cross validation and all the four methods above use the same groups of training and test set for fair evaluation. As mentioned earlier, both Smalter’s method and Xu’s method directly treat *U* as negative set. ProDiGe uses its bagging method to choose random subsets *RS* from *U* and aggregate all the individual classifiers built using *P* and different *RS*. Our PUDI method partitions *U* into four label sets and then builds a multi-level weighted SVM classifier that takes the confidence levels of these label sets into consideration.

Table 1 shows that our proposed PUDI method is able to achieve 76.5% F-measure which is 14.2, 15.1 and 2.0% better than Smalter’s method, Xu’s method (KNN with *K* = 5) and ProDiGe method, respectively. Particularly, compared with ProDiGe, our PUDI method achieves similar precision but 5.1% higher recall, indicating that our multi-level PUDI method can better handle the unlabelled data *U* for identifying the hidden disease genes in the test set. For Xu’s method, we increased its K value from 1 to 21, but its F-measure only changes slightly, ranging from 61.2 to 61.5. The experimental results in Table 1 confirm the benefits of appropriately processing the unknown gene set *U*.

**Table 1.** Overall comparison among different techniques

Techniques	Precision (*p*) (%)	Recall (*r*) (%)	F-measure (*F*) (%)
PUDI	72.3	81.0	76.5
ProDiGe	72.4	75.9	74.5
Smalter’s method	62.9	61.5	62.2
Xu’s method (1)	65.0	55.6	59.9
Xu’s method (5)	66.3	57.1	61.3

Recall that we chose those disease genes with at least two-thirds non-zero features since they can provide sufficient informative information for classifiers building. To further evaluate the generalization ability of PUDI, we constructed 10 new test sets which consist of all the 121 *poorly annotated* disease genes and 10 groups of randomly selected 121 unlabelled genes (both with less than two-thirds non-zero features). Interestingly, we observed that PUDI, in average, achieves 86.5% F-measure, indicating that PUDI classifier is robust enough to accurately identify those poorly annotated disease genes by automatically choosing those highly distinguishing biological features.

Second, we conducted an experiment to investigate the effectiveness of the individual feature category and their combinations, as shown in Table 2 (Rows 2–6 and 7–11, respectively). Among the five individual categories, using only the BP ontology achieves the highest F-measure (71.3%), higher than the other feature categories where they have higher recalls but much lower precisions. Further, we filtered out one category from the combined feature set each time. The results in Rows 7–11 showed that using a combined feature set without PPI category can gain better performance than those of other four kinds of combined feature groups. This is probably because we only have four PPI features, so removing them will only affect the classification performance slightly. Note the performance of using a combined feature set without protein domains leads to the worst performance, indicating protein domains, as proteins’ evolutionarily conserved modules, are useful for identifying disease genes. The performance of using all the features (Table 1) is still the best, confirming that integrating all the available biological resources is very valuable for disease gene prediction task.

**Table 2.** Results of individual feature and combinations of features

Category	Precision (*p*) (%)	Recall (*r*) (%)	F-measure (*F*) (%)
BP	63.4	81.3	71.3
MF	50.3	99.6	68.6
CC	54.5	93.5	67.8
D	56.2	86.5	68.1
PPI	55.1	88.2	67.8
ALL-BP	65.3	83.3	73.2
ALL-MF	66.0	84.7	74.2
ALL-CC	67.4	85.7	75.4
ALL-D	62.3	86.9	72.6
ALL-PPI	67.9	86.7	76.1

Third, we performed a sensitivity study on the parameters used in the PUDI algorithm, namely, *N* (used in our feature selection method to control the number of features for *MF*, *BP*, *CC* and *D*), *Q* (decides the number of neighbors used in gene similarity network) and *α* (used in Random Network to decide how much the influence flows back to initial nodes). Please refer to Supplementary Tables S4–S6 for detailed discussion. These results showed that PUDI was insensitive to the specific values of *N* and *Q.* In addition, the best performance was obtained when *α* = 0.8 which coincided with the recommended value by (Li and Patra, 2010).

Fourth, we investigated the capability of our proposed algorithm to detect disease genes for *specific disease classes*/*groups*—this is much more practically useful than predict *general* disease genes, e.g. developing novel drugs to tackle disease genes associated with a specific disease for pharmaceutical industry. In this work, we chose all disease classes (Goh *et al.*, 2007) which have at least 20 confirmed disease genes and we obtained 8 *specific disease classes* in total. Here we listed the results for cardiovascular diseases and endocrine diseases. The results for the other six disease classes are listed in Supplementary Table S7. For the two disease classes, we selected the disease genes containing the title ‘cardiovascular’ or ‘endocrine’ in the causative disease phenotype descriptions from GENECARD and OMIM. A total of 107 cardiovascular disease genes and 81 endocrine disease genes are collected, respectively (both treated as positive set *P*). Then, 10 groups of unlabeled gene sets are randomly selected from all gene set as the 10 unlabeled sets *U* (*U* has the same size with *P*, i.e. |*P*| = |*U*|). Again, all the approaches are evaluated on the identical groups of test data. Given that we have relatively small number of disease genes, to avoid tiny partitions, we performed 3-fold cross validation for each of the 10 training groups and reported the average results in Table 3.

**Table 3.** Cardiovascular and endocrine disease gene classification

Disease class	Techniques	Precision (*p*) (%)	Recall (*r*) (%)	F-measure (*F*) (%)
Cardiovascular diseases	PUDI	82.0	80.6	80.4
	ProDiGe	54.3	96.3	69.3
	Smalter’s method	75.4	67.6	70.6
	Xu’s method (1)	72.1	60.0	65.4
	Xu’s method (5)	73.6	63.0	67.9
Endocrine diseases	PUDI	83.6	75.3	79.2
	ProDiGe	57.3	87.7	69.3
	Smalter’s method	76.4	58.8	66.5
	Xu’s method (1)	75.4	62.0	68.0
	Xu’s method (5)	72.5	62.2	67.0

Table 3 shows that our proposed PUDI algorithm is 9.8 and 9.9% better than the best results from Smalter’s method, Xu’s method and ProDiGe method for cardiovascular and endocrine diseases, respectively. For Xu’s method, we have also tried different K valued from 1 to 21. It achieved the best results 72.1% with *K* = 17 for cardiovascular disease and 68.0% with *K* = 1 for endocrine disease in terms of F-measure.

We observed ProDiGe performs 1.3% worse than Smalter’s method for cardiovascular disease but 1.3–2.8% better than Xu’s method and Smalter’s method for endocrine diseases, showing that it cannot achieve consistently better results than other methods. As we mentioned earlier, since the subsets *RS* that are randomly selected from *U* may still contain unknown disease genes, it will affect the performance of individual classifiers built using *P* and *RS* as well as the final aggregated classifier. On the other hand, our proposed PUDI method partitions *U* into four label sets, so that the multi-level weighted SVM classifier, can better exploit *U* as training sets by taking the varying confidence levels of the training sets into consideration. The results on six other disease groups shown in Supplementary Table S7 also demonstrate that PUDI is much more accurate than the other state-of-the-art techniques. To further evaluate the prediction performance among different techniques, the ROC curves on all the eight disease groups are provided in Supplementary Figure S1, indicating PUDI outperforms other techniques significantly.

Finally, we applied PUDI for uncovering *novel* disease genes. This is different from the evaluations above where we performed cross validations, i.e. we used part of the confirmed disease genes as the positive training set and the remaining confirmed disease genes as positive test set. Here, we attempted to discover putative disease genes that are not presented in the current confirmed disease gene data set. In other words, we will exploit all the confirmed disease genes to predict novel disease genes. As a case study, we applied our PUDI algorithm to discover novel disease genes for cardiovascular diseases. Our algorithm detected 10 unlabeled genes that were not in benchmark/confirmed disease gene data set. We then performed literature search to check if any of these putative disease genes predicted is indeed associated to cardiovascular diseases. We found that four of the predicted disease genes, namely, ATF4, MBNL1, NCKAP1 and CXCL14, have been reported to be related to cardiovascular diseases. For ATF4, it has been verified to play an important role in cardiovascular diseases using reverse transcription/real-time polymerase chain reaction and western blotting (Afonyushkin *et al.*, 2010). For MBNL1, it exhibited a regionally restricted pattern of expression in canal region endocardium and ventricular myocardium during endocardial cushion development in chicken (Vajda *et al.*, 2009). Also, mutations of NCKAP1 showed specific morphogenetic defects: these mouse failed to close the neural tube, also failed to form a single tube (cardia bifida) and showed delayed migration of endoderm and mesoderm (Rakeman *et al.*, 2006). In addition, for CXCL14, it enhanced the insulin-induced tyrosine phosphorylation of insulin receptors and insulin receptor substrate-1, suggesting that CXCL14 played a causal role in high-fat diet-induced obesity, which was frequently associated with hypertension (one type of cardiovascular diseases) (Takahashi *et al.*, 2007).

We also applied PUDI algorithm to detect *novel* endocrine disease genes. Please refer to Section 5 in Supplementary Material.

Furthermore, we performed our PUDI algorithm using all the confirmed disease genes as positive training set *P* (not focus on one specific disease). We predicted 1110 novel disease genes and we selected the top 20 genes based on their SVM probabilities (we transformed the outputs from SVM into probabilities). Based on the literature search, the results in Table 4 show that 14 out of 20 (70%) predicted disease genes are indeed associated with one or more diseases (references are listed in Supplementary Material).

**Table 4.** Predicted novel disease genes using all confirmed genes

Genes	Prob (%)	Relevant disease	References
GP5	99.2	Bernard–Soulier syndrome	(Roth *et al.*, 1990)
		Gray platelet syndrome	(Berger *et al.*, 1996)
		Platelet disorder	(Shi *et al.*, 2004)
		Autoimmune thrombocytopenia	(Mayer *et al.*, 1996)
		Coagulopathy	(Modderman *et al.*, 1992)
		Thrombocytopenia	(Acar *et al.*, 2008)
		Thrombosis	(Ravanat *et al.*, 1997)
ALG13	97.9		
ADPRHL1	96.7		
PARVA	96.6	Tumors	(Attwell *et al.*, 2003)
		Cancer	(Sepulveda *et al.*, 2006)
ODAM	96.4		
ANGPTL1	96.3	Melanoma	(Smagur *et al.*, 2005)
		Tumors	(Xu *et al.*, 2004)
PTK7	96.1	Panic	(Eser *et al.*, 2005)
		Panic attacks	(van Megen *et al.*, 1997)
		Panic disorder	(Bradwejn *et al.*, 1992)
		Premenstrual dysphoric disorder	(Le Mellédo *et al.*, 1999)
		Effects cardiovascular	(Bradwejn *et al.*, 1994)
		Agoraphobia	(Koszycki *et al.*, 1996)
		Anxiety disorders	(Bradwejn *et al.*, 1990)
		Colon carcinoma	(Mossie *et al.*, 1995)
WSB1	95.7	neurobalstoma	(Chen, 2006)
AFF1	95.0	Lymphoblastic leukemia acute	(Bertrand *et al.*, 2001)
		Acute leukemia	(Chen *et al.*, 1993)
		Leukemogenesis	(Yamamoto *et al.*, 1998)
		Leukemia	(Li *et al.*, 1998)
		Chromosomal aberrations	(Nakamura *et al.*, 1993)
INHBB	94.7	Tumors	(Peschon *et al.*, 1992)
MAPK12	94.4	Shock	(Cuenda *et al.*, 1997)
PHLDA1	94.3	Tumors	(Nagai *et al.*, 2007)
CABLES2	94.0		
BDH2	94.0		
CD97	94.0	Thyroid carcinoma	(Hoang-Vu *et al.*, 1999)
		Thyroid carcinoma anaplastic	(Hoang-Vu *et al.*, 1999)
		Arthritis reactive	(Hamann *et al.*, 1999)
		Colorectal tumors	(Steinert *et al.*, 2002)
		Colorectal carcinoma	(Steinert *et al.*, 2002)
SLC29A4	93.9		
FAIM	93.8	Leukemia, lymphocytic, Acute	(Ross *et al.*, 2003)
EIF2AK2	93.8	Virus infection	(Gil *et al.*, 2000)
		Vesicular stomatitis	(Lee *et al.*, 1996)
		Hepatitis c	(Hiasa *et al.*, 2003)
		Influenza	(Min *et al.*, 2007)
		Herpes simplex	(Smith *et al.*, 2006)
KRT20	93.7	Carcinoma merkel cell	(Cheuk *et al.*, 2001)
		Carcinoma mucinous	(Ji *et al.*, 2002)
		adenocarcinoma	(Chen *et al.*, 2004)
ITGB1BP2	93.7	Cardiac hypertrophy	(Brancaccio *et al.*, 2003)
		hypertrophy	(Palumbo et al., 2009)

Detailed discussions on the computational efficiency of all the four related algorithms (PUDI, ProDiGe, Smalter’s method and Xu’s method) can be found in Section 7 of the Supplementary Material.

## 4 CONCLUSIONS

To identify disease genes, traditional machine learning methods typically build a binary classification model using confirmed disease genes as positive set *P* and unknown genes as negative set *N*. The negative set *N* is noisy because the unknown gene set *U* contains some unknown disease genes. As such, the classifiers built do not perform as well as they could have.

In this work, we have proposed a novel PU learning approach PUDI for disease gene prediction. We introduced a new feature selection method to identify the discriminating features and performed a further partitioning of the unlabeled set *U* into multiple training sets for a more refined treatment of *U* to build the final classifier. We found that PUDI could better model the classification problem for disease gene prediction as it achieved significantly better results than the state-of-the-art methods. Given that many machine learning problems in biomedical research do involve positive and unlabeled data instead of negative data, we believe that the performance of machine learning methods for these problems can possibly be further improved by adopting a PU learning approach (Cerulo, *et al.*, 2010; Mordelet *et al.*, 2008), as we have done here for disease gene identification. For future work, we will consider to integrate more biological resources (Linghu *et al.*, 2009), such as gene expression data, etc. In addition, we may explore more complicated machine learning methods to better model the positive and unlabelled data distributions.

*Funding*: This research was supported by Singapore MOE AcRF Grant No: MOE2008-T2-1-074.

*Conflict of Interest:* none declared.

## Supplementary Material

Supplementary Data
