# Supplementary material for: Positive-unlabeled learning for disease gene identification
Source: Bioinformatics. 2012 Aug 24;28(20):2640–7. doi: 10.1093/bioinformatics/bts504 (PMC3467748; doi:10.1093/bioinformatics/bts504)
Supplement: Supplementary Data [file supp_28_20_2640__index.html]

Positive-unlabeled learning for disease gene identification — Supplementary Data 

# Positive-unlabeled learning for disease gene identification

## Supplementary Data

files

**Files in this Data Supplement:**

- Supplementary Data - docx file
