# Supplementary material for: Positive-unlabeled learning for disease gene identification
Source: Bioinformatics. 2012 Aug 24;28(20):2640–7. doi: 10.1093/bioinformatics/bts504 (PMC3467748; doi:10.1093/bioinformatics/bts504)
Supplement: Supplementary Data [file supp_bts504_Bioinformatics_SupplementaryFile_Submission.docx]

**Supplementary Materials**

1. **Features and Feature Selection**

**Table S.1 The feature numbers for BP, MF, CC and D**

|  | **BP** | **MF** | **CC** | **D** |
| --- | --- | --- | --- | --- |
| Number of features | 21302 | 9857 | 2949 | 13672 |

BP: biological processes, MF: molecular functions, CC: cellular components, D: protein domains. This table shows there are huge numbers of features in category BP, MF, CC and D.

As mentioned in Section 2.2, we have proposed a novel feature selection method to choose GO features and domain features. The following Table S.2 and S.3 listed some highly distinguishing Go features (BP, MF, CC) and domains respectively.

**Table S.2 The distinguishing features for BP, MF and CC**

| **GO term** | **GO** | **Definition** | **Disease Gene Number** |
| --- | --- | --- | --- |
| GO:0007165 | BP | signal transduction; signaling cascade | 389 |
| GO:0050896 | BP | Response to stimulus; Physiological response to stimulus | 172 |
| GO:0007166 | BP | Cell surface receptor signaling pathway; Cell surface receptor linked signaling pathway; | 89 |
| GO:0035556 | BP | Intracellular signal transduction; Intracellular signaling cascade | 64 |
| GO:0000166 | MF | Nucleotide binding | 538 |
| GO:0008134 | MF | Transcription factor binding | 101 |
| GO:0019899 | MF | Enzyme binding | 89 |
| GO:0016020 | CC | Membrane | 782 |
| GO:0005634 | CC | Nucleus; cell nucleus | 1146 |

Below, we will introduce some of the distinguishing GO features and their relationships to certain diseases.

**GO:007165 (signal transduction)**: according to website: <http://signaling.unibas.ch/research/signal-transduction>, sensing of environments at the cellular level relies on signal transduction; many disease processes, such as diabetes and heart disease arise from defects in these pathways, playing crucial rules in biology and medicine).

**GO:0007166 (Cell surface receptor signaling pathway)**: The pathway begins with the binding of extracellular signaling molecules to the cell surface receptors, ends with regulation of a downstream cellular process. Many diseases are caused by membrane receptor function disorder due to deficiency or disorder of the receptor. Recent research has found that the cell surface receptor TM4SF5 has biological functions of the migration ability of hepatic cells and hepatoma, and that the cortical NMDA receptor properties and membrane fluidity are altered in Alzheimer’s disease. (Scheuer, K. et al. 1996).

**GO:0000166 (Nucleotide binding)**: Proteins which bind a nucleotide, a phosphate ester of a nucleoside consisting of a purine or pyrimidine base linked to ribose or deoxyribose phosphates. G proteins function as molecular switches. When they bind guanosine triphosphate (GTP), a type of nucleotide, they are 'on', and, when they bind guanosine diphosphate (GDP), they are 'off'. Therefore, nucleotide binding is crucial to the activity of G protein. G proteins are important [signal transducing](http://en.wikipedia.org/wiki/Signal_transducing) molecules in cells. Malfunction of GPCR [G Protein-Coupled Receptor] signaling pathways are involved in many diseases, such as [diabetes](http://en.wikipedia.org/wiki/Diabetes), blindness, allergies, depression, cardiovascular defects, and certain forms of [cancer](http://en.wikipedia.org/wiki/Cancer) ([Wu, G.](http://en.wikipedia.org/w/index.php?title=Ge_Wu&action=edit&redlink=1) 2010).

**GO:0008134 (Transcription factor binding)**: A transcription factor (a sequence-specific DNA-binding factor) is a protein that binds to specific DNA sequences, thereby controlling the flow of genetic information from DNA to mRNA. DNA-binding domain is an important identity of transcription factor. Disorder of a transcription factor can arise to deficiency the function of transcription factor binding. Many transcription factors are either [tumor suppressors](http://en.wikipedia.org/wiki/Tumor_suppressor) or [oncogenes](http://en.wikipedia.org/wiki/Oncogene), thus, mutations or aberrant regulations of them are associated with cancer. Three groups of transcription factors are known to be important in human cancer: (1) the [NF-kappaB](http://en.wikipedia.org/wiki/NF-kappaB) and [AP-1](http://en.wikipedia.org/wiki/AP-1_transcription_factor) families, (2) the [STAT](http://en.wikipedia.org/wiki/STAT_protein) family and (3) the [steroid receptors](http://en.wikipedia.org/wiki/Steroid_hormone_receptor) (Libermann, T.A. et al. 2006).

**GO:0016020(membrane)**: Many diseases involve the membrane to some degrees. Phospholopids are crucial to our cell membranes. Phospholopid altercations, caused by the oxidative stress arise by Alzheimer’s disease, would compromise cell membrane, leading to malfunction of brain cells. Duchnee Muscular Dystrophy affects dystrophin in the muscle cell. Without dystrophin, the cell membrane would be not able to repair itself, therefore destroy the muscle cell and lead to Duchnee Muscular Dystrophy.

Table S.3 listed those protein domains which are highly related to the disease and corresponding disease genes.

**Table S.3 The distinguishing features for domain D**

| **Domain** | **Disease name** | **Disease gene** |
| --- | --- | --- |
| **PF00096** | HYPERTROPHIC NEUROPATHY OF DEJERINE-SOTTAS: 145900;  NEUROPATHY,CONGENITAL HYPOMYELINATING: 605253;  CHARCOT-MARIE-TOOTH DISEASE, DEMYELINATING, TYPE 1D; CMT1D: 607678 | EGR2 |
|  | NEUTROPENIA, NONIMMUNE CHRONIC IDIOPATHIC, OF ADULTS: 607847 | GFI1 |
|  | PALLISTER-HALL SYNDROME; PHS:146510;  POLYDACTYLY, POSTAXIAL, TYPE A1:174200;  POLYDACTYLY, PREAXIAL IV:174700;  GREIG CEPHALOPOLYSYNDACTYLY SYNDROME; GCPS: 175700;  HYPOTHALAMIC HAMARTOMAS CONGENITAL HYPOTHALAMIC HAMARTOMA SYNDROME, INCLUDED; CHHS, INCLUDED: 241800 | GLI3 |
|  | GASTRIC CANCER:137215;PROSTATE CANCER:176807 | KLF6 |
|  | SALIVARY GLAND ADENOMA, PLEOMORPHIC": 181030 | PLAG1 |
|  | DIABETES MELLITUS, TRANSIENT NEONATAL, 1: 601410 | PLAGL1 |
|  | TOWNES-BROCKS SYNDROME; TBS:107480 | SALL1 |
|  | IVIC SYNDROME:147750;  DUANE-RADIAL RAY SYNDROME; DRRS:607323 | SALL4 |
|  | PIEBALD TRAIT; PBT:172800;  WAARDENBURG SYNDROME, TYPE IID:608890 | SNAI2 |
|  | TRICHORHINOPHALANGEAL SYNDROME, TYPE I; TRPS1:190350;  TRICHORHINOPHALANGEAL SYNDROME, TYPE III; TRPS3:190351 | RPS1 |
|  | FRASIER SYNDROME:136680;  MESOTHELIOMA, MALIGNANT:156240;  WILMS TUMOR 1; WT1:194070;  DENYS-DRASH SYNDROME; DDS:194080;  NEPHROTIC SYNDROME, EARLY-ONSET, WITH DIFFUSE MESANGIAL SCLEROSIS:256370;  MEACHAM SYNDROME:608978 | WT1 |
|  | MOWAT-WILSON SYNDROME:235730 | ZEB2 |
|  | PROSTATE CANCER:176807  PTOSIS, HEREDITARY CONGENITAL 1; PTOS1:178300 | ZFHX3 |
|  | DIABETES MELLITUS, TRANSIENT NEONATAL, 1: 601410 | ZFP57 |
|  | TETRALOGY OF FALLOT:187500 | ZFPM2 |
|  | HETEROTAXY, VISCERAL, 1, X-LINKED; HTX1: 306955 | ZIC3 |
| **PF00001** | RESTING HEART RATE:607276 | ADRB1 |
|  | ASTHMA, SUSCEPTIBILITY TO:600807  OBESITY LEANNESS, INCLUDED:601665 | ADRB2 |
|  | OBESITY LEANNESS, INCLUDED:601665 | ADRB3 |
|  | HYPERTENSION, ESSENTIAL:145500  RENAL TUBULAR DYSGENESIS; RTD:267430 | AGTR1 |
|  | DIABETES INSIPIDUS, NEPHROGENIC, X-LINKED: 304800 | AVPR2 |
|  | WHIM SYNDROME:193670 | CXCR4 |
|  | MYOCLONIC DYSTONIA:159900 | DRD2 |
|  | SCHIZOPHRENIA; SCZD:181500  TREMOR, HEREDITARY ESSENTIAL, 1; ETM1:190300 | DRD3 |
|  | ATTENTION DEFICIT-HYPERACTIVITY DISORDER; ADHD:143465  NOVELTY SEEKING PERSONALITY TRAIT:601696 | DRD4 |
|  | ATTENTION DEFICIT-HYPERACTIVITY DISORDER; ADHD:143465  BLEPHAROSPASM, BENIGN ESSENTIAL:606798 | DRD5 |
|  | MIGRAINE WITH OR WITHOUT AURA, SUSCEPTIBILITY TO, 1: 157300 | EDNRA |
|  | WAARDENBURG-SHAH SYNDROME:277580  HIRSCHSPRUNG DISEASE, SUSCEPTIBILITY TO, 2; HSCR2:600155  ABCD SYNDROME:600501 | EDNRB |
|  | OVARIAN DYSGENESIS 1; ODG1:233300  TWINNING, DIZYGOTIC OVARIAN RESPONSE TO FSH STIMULATION, INCLUDED:276400  OVARIAN HYPERSTIMULATION SYNDROME:608115 | FSHR |
|  | SHORT STATURE, IDIOPATHIC, AUTOSOMAL:604271 | GHSR |
|  | FERTILE EUNUCH SYNDROME:228300 | GNRHR |
|  | ALCOHOL DEPENDENCE:103780  OBSESSIVE-COMPULSIVE DISORDER 1; OCD1:164230  SCHIZOPHRENIA; SCZD:181500  ANOREXIA NERVOSA, SUSCEPTIBILITY TO, 1; ANON1:606788  MAJOR DEPRESSIVE DISORDER; MDD:608516 | HTR2A |
|  | HYPOGONADOTROPIC HYPOGONADISM:146110  PRECOCIOUS PUBERTY, CENTRAL:176400 | KISS1R |
|  | PRECOCIOUS PUBERTY, MALE-LIMITED:176410  HYPERGONADOTROPIC HYPOGONADISM; HHG: 238320 | LHCGR |
|  | WOOLLY HAIR, AUTOSOMAL RECESSIVE:278150 | LPAR6 |
|  | ALBINISM, OCULOCUTANEOUS, TYPE II; OCA2: 203200  SKIN/HAIR/EYE PIGMENTATION, VARIATION IN, 2; SHEP2: 266300 | MC1R |
|  | GLUCOCORTICOID DEFICIENCY 1; GCCD1:202200 | MC2R |
|  | MYCOBACTERIUM TUBERCULOSIS, SUSCEPTIBILITY TO MYCOBACTERIUM TUBERCULOSIS, PROTECTION AGAINST, INCLUDED: 607948 | MC3R |
|  | OBESITY LEANNESS, INCLUDED:601665 | MC4R |
|  | ASTHMA-RELATED TRAITS, SUSCEPTIBILITY TO, 2: 608584 | NPSR1 |
|  | COLORBLINDNESS, BLUE-MONO-CONE-MONOCHROMATIC TYPE; CBBM: 303700  COLORBLINDNESS, PARTIAL, PROTAN SERIES; CBP: 303900 | OPN1LW |
|  | TRITANOPIA:190900 | OPN1SW |
|  | KALLMANN SYNDROME 3; KAL3:244200 | PROKR2 |
|  | ASTHMA-RELATED TRAITS, SUSCEPTIBILITY TO, 1:607277 | PTGDR |
|  | ASTHMA, NASAL POLYPS, AND ASPIRIN INTOLERANCE: 208550 | PTGER2 |
|  | FUNDUS ALBIPUNCTATUS RETINITIS PUNCTATA ALBESCENS, INCLUDED:136880 | RHO |
|  | PITUITARY ADENOMA, GROWTH HORMONE-SECRETING:102200 | SSTR5 |
|  | HYPOGONADOTROPIC HYPOGONADISM:146110 | TACR3 |
| **PF00076** | Neuroepithelioma:612219 | EWSR1 |
|  | AMYOTROPHIC LATERAL SCLEROSIS 6:608030 | FUS |
|  | DIABETES MELLITUS, NONINSULIN-DEPENDENT; NIDDM:125853 | IGF2BP2 |
|  | OCULOPHARYNGEAL MUSCULAR DYSTROPHY; OPMD:164300 | PABPN1 |
|  | OBESITY LEANNESS, INCLUDED:601665 | PPARGC1B |
|  | TARP SYNDROME; TARPS:311900 | RBM10 |
|  | Alopecia, neurologic defects, and endocrinopathy syndrome:612079 | SART3 |
|  | POROKERATOSIS, DISSEMINATED SUPERFICIAL ACTINIC, 1; DSAP1:175900 |  |
|  | Chondrosarcoma, extraskeletal myxoid:612237 | TAF15 |
|  | Frontotemporal lobar degeneration, TARDBP-related:612069 | TARDBP |
| **PF00069** | FIBRODYSPLASIA OSSIFICANS PROGRESSIVA; FOP:135100 | ACVR1 |
|  | OSLER-RENDU-WEBER SYNDROME 2; ORW2:600376 | ACVRL1 |
|  | BREAST CANCER:114480  COLORECTAL CANCER; CRC:114500 :167000  PROTEUS SYNDROME:176920  SCHIZOPHRENIA; SCZD:181500 | AKT1 |
|  | DIABETES MELLITUS, NONINSULIN-DEPENDENT; NIDDM:125853  HYPOGLYCEMIA, NEONATAL, SIMULATING FOETOPATHIA DIABETICA:240900 | AKT2 |
|  | "PERSISTENT MULLERIAN DUCT SYNDROME, TYPES I AND II; PMDS":261550 | AMHR2 |
|  | COLORECTAL CANCER; CRC:114500 | AURKA |
|  | JUVENILE POLYPOSIS SYNDROME; JPS:174900 | BMPR1A |
|  | BRACHYDACTYLY, TYPE A2; BDA2:112600 | BMPR1B |
|  | PULMONARY HYPERTENSION, PRIMARY; PPH1:178600 | BMPR2 |
|  | CARDIOFACIOCUTANEOUS SYNDROME:115150  LUNG CANCER:211980 :613706 | BRAF |
|  | FG SYNDROME 4; FGS4:300422 | CASK |
|  | ANGELMAN SYNDROME; AS:105830: 300672  BREAST CANCER:114480  PROSTATE CANCER:176807  OSTEOGENIC SARCOMA:259500 | CHEK2 |
|  | DYSTROPHIA MYOTONICA 1:160900 | DMPK |
|  | EPIPHYSEAL DYSPLASIA, MULTIPLE, WITH EARLY-ONSET DIABETES MELLITUS:226980 | EIF2AK3 |
|  | LEBER CONGENITAL AMAUROSIS, TYPE I; LCA1: 204000 | GUCY2D |
|  | IRAK4 DEFICIENCY:607676 | IRAK4 |
|  | PARKINSON DISEASE 8; PARK8:607060 | LRRK2 |
|  | CARDIOFACIOCUTANEOUS SYNDROME:115150 | MAP2K1  MAP2K2 |
|  | LUNG CANCER:211980 | MAP3K8 |
|  | EPILEPTIC ENCEPHALOPATHY, LENNOX-GASTAUT TYPE:606369 | MAPK10 |
|  | THROMBOCYTOPENIA 2; THC2:188000 | MASTL |
|  | CARDIOMYOPATHY, FAMILIAL HYPERTROPHIC; CMH: 192600 | MYLK2 |
|  | DEAFNESS, AUTOSOMAL RECESSIVE 30; DFNB30: 607101 | MYO3A |
|  | ACROMESOMELIC DYSPLASIA, MAROTEAUX TYPE; AMDM ST. HELENA DYSPLASIA, INCLUDED:602875; | NPR2 |
|  | SPINOCEREBELLAR ATAXIA 14; SCA14:605361 | PRKCG |
|  | STROKE, ISCHEMIC:601367 | PRKCH |
|  | COFFIN-LOWRY SYNDROME; CLS:303600 | RPS6KA3 |
|  | PEUTZ-JEGHERS SYNDROME; PJS:175200  TESTICULAR TUMORS:273300 | STK11 |
|  | MULTIPLE SELF-HEALING SQUAMOUS EPITHELIOMA; MSSE: 132800  LOEYS-DIETZ SYNDROME, TYPE 2A; LDS2A:608967 | TGFBR1 |
|  | ESOPHAGEAL CANCER ESOPHAGEAL SQUAMOUS CELL CARCINOMA, INCLUDED; ESCC, INCLUDED: 133239 | TGFBR2 |
|  | SPINOCEREBELLAR ATAXIA 11; SCA11:604432 | TTBK2 |
|  | TIBIAL MUSCULAR DYSTROPHY, TARDIVE:600334  HEREDITARY MYOPATHY WITH EARLY RESPIRATORY FAILURE; HMERF: 603689  CARDIOMYOPATHY, DILATED, 1G; CMD1G:604145  MUSCULAR DYSTROPHY, LIMB-GIRDLE, TYPE 2J; LGMD2J:608807 | TTN |
|  | PSEUDOHYPOALDOSTERONISM, TYPE II; PHA2:145260  NEUROPATHY, HEREDITARY SENSORY AND AUTONOMIC, TYPE II; HSAN2: 201300 | WNK1 |
|  | PSEUDOHYPOALDOSTERONISM, TYPE II; PHA2:145260 | WNK4 |
| **PF07686** | OSTEOCHONDRITIS DISSECANS; OD:165800  SPONDYLOEPIPHYSEAL DYSPLASIA, KIMBERLEY TYPE:608361 | ACAN |
|  | CD8 DEFICIENCY, FAMILIAL:608957 | CD8A |
|  | C SYNDROME:211750 | CD96 |
|  | HASHIMOTO THYROIDITIS:140300 | CTLA4 |
|  | IgE RESPONSIVENESS, ATOPIC; IGER:147050 | HAVCR1 |
|  | DYSSEGMENTAL DYSPLASIA, SILVERMAN-HANDMAKER TYPE; DDSH: 224410  SCHWARTZ-JAMPEL SYNDROME, TYPE 1; SJS1:255800 | HSPG2 |
|  | HEMANGIOMA, CAPILLARY INFANTILE:602089 | KDR |
|  | CHARCOT-MARIE-TOOTH DISEASE, DEMYELINATING, TYPE 1B; CMT1B: 118200  HYPERTROPHIC NEUROPATHY OF DEJERINE-SOTTAS: 145900  ROUSSY-LEVY HEREDITARY AREFLEXIC DYSTASIA: 180800  NEUROPATHY, CONGENITAL HYPOMYELINATING: 605253  CHARCOT-MARIE-TOOTH DISEASE, AXONAL, TYPE 2I: 607677  CHARCOT-MARIE-TOOTH DISEASE, AXONAL, TYPE 2J: 607736  CHARCOT-MARIE-TOOTH DISEASE, DOMINANT INTERMEDIATE D: 607791 | MPZ |
|  | NEPHROSIS 1, CONGENITAL, FINNISH TYPE; NPHS1:256300 | NPHS1 |
|  | MULTIPLE SCLEROSIS, SUSCEPTIBILITY TO; MS:126200  SYSTEMIC LUPUS ERYTHEMATOSUS, SUSCEPTIBILITY TO, 2; SLEB2: 605218 | PDCD1 |
|  | POLYCYSTIC LIPOMEMBRANOUS OSTEODYSPLASIA WITH SCLEROSING LEUKOENCEPHALOPATHY; PLOSL: 221770 | TREM2 |
|  | WAGNER SYNDROME 1; WGN1:143200 | VCAN |

1. **Sensitivity analysis of parameters in PUDI algorithm**

We perform a sensitivity study for all the three parameters used in the algorithm, i.e. parameter N (used in our feature selection method to control the number of features from MF, BP, CC and D), parameter Q (decides the number of neighbors used in our gene similarity network) and parameter $\alpha$ (used in Random Network to decide how much the influence flows returning back to initial nodes).

Recall that we have one parameter N in our feature selection method to control the number of features from *MF, BP, CC* and *D*. To study the effect of parameter *N* on the performance of our algorithm, we run our method with *N* from 500 to 2000 with step 500. The results are shown in **Table S.4.** The performance is improved with increasing value of *N* from 500 to 1000, indicating that incorporating more features is helpful for classifying target disease genes. However, if we further include more features with low feature discrimination scores (say N=2000), noisy features will be included and eventually affect the performance of disease gene classification.

**Table S.4** Effect of parameter N (in feature selection) to classification performance

| **# Parameter *N*** | **Precision (*p*)** | **Recall(*r*)** | **F-measure (*F*)** |
| --- | --- | --- | --- |
| 500 | 70.8% | 82.5% | 76.2% |
| 1000 | 72.4% | 81.0% | 76.5% |
| 1500 | 70.2% | 81.7% | 76.2% |
| 2000 | 69.9% | 82.0% | 75.5% |

To study the effect of the parameter Q, we run our algorithm with Q from 3 to 9 while fixing N = 1000. Results are shown in Table S.5. The F-measure is slightly decreased with the value of Q from 5 to 9, indicating that incorporating more edges with relatively low similarities may introduce the noisy connections and thus affect the performance of disease gene identification. Nevertheless, the performance with parameter Q from 3 to 9 without very slight difference suggests that our algorithm is robust to the noisy gene connections and insensitive to the specific value of Q.

**Table S.5** Effect of parameter Q (in constructing gene similarity network) to classification performance

| **Parameter *Q*** | **Precision (*p*)** | **Recall(*r*)** | **F-measure (*F*)** |
| --- | --- | --- | --- |
| 3 | 71.9% | 81.3% | 76.3% |
| 4 | 72.2% | 81.0% | 76.3% |
| 5 | 72.4% | 81.0% | 76.5% |
| 6 | 72.5% | 80.7% | 76.4% |
| 7 | 72.0% | 80.8% | 76.2% |
| 8 | 72.3% | 80.3% | 76.1% |
| 9 | 72.6% | 80.1% | 76.2% |

Parameter $\alpha$ in random walk algorithm is used to control how much the influence flows returning back to initial nodes (Genes in P and RN) at each iteration of the algorithm. In addition, it is also used to be to judge unlabeled genes assigned to likely positive *LP* or likely negative *LN*. With a large $\alpha$ in random walk algorithm, the flows are likely to return to the seed nodes. Therefore the nodes near to seeds are likely to gain higher scores to be assigned to set *LP/LN*. On the contrary, with a small $\alpha$ in random walk algorithm, the flows are likely to flow out of the seed nodes and spread to nodes far away from seeds, therefore those nodes near to seeds are likely to gain relatively lower scores to be assigned to weak negative set *WN*. When fixing parameters *N* = 1000, *K* = 5, we are able to obtain higher F-measure value with increasing value of $\alpha$, as shown in **Table S.6**. Biologically, this is reasonable since unlabeled genes which share various biological evidences with labeled ones more likely belong to same class, either disease genes or non-disease genes.

**Table S.6** Effect of parameter $\alpha$ (in random network propagation) to classification performance

| **Parameter** $\boldsymbol{\alpha}$ | **Precision (*p*)** | **Recall(*r*)** | **F-measure (*F*)** |
| --- | --- | --- | --- |
| 0.6 | 66.5% | 82.3% | 73.5% |
| 0.7 | 70.4% | 82.5% | 76.0% |
| 0.8 | 72.4% | 81.0% | 76.5% |
| 0.9 | 73.0% | 79.7% | 76.2% |

1. **Parameter Setting of Multi-level example learning in Weighted SVM**

For Multi-level SVM, we set its penalty factors in following way: let SVM penalty factors $c=c_{-}^{'''}$, and $w_{+}^{'}=c_{+}^{'}/c_{-}^{'''}$, $w_{+}^{''}=c_{+}^{''}/c_{-}^{'''}$, $w_{-}^{'}=c_{-}^{'}/c_{-}^{'''}$, $w_{-}^{''}=c_{-}^{''}/c_{-}^{'''}$, then we can get an optimizing goal function using following formula:

$$\frac{1}{2}\left\| w \right\|^{2}+c(w_{+}^{'}\sum_{i\in P} \xi_{i}+w_{+}^{''}\sum_{i\in LP} \xi_{i}+w_{-}^{'}\sum_{i\in RN} \xi_{i}+{w'}_{-}^{'}\sum_{i\in LN} \xi_{i}+\sum_{i\in WN} \xi_{i})$$

Subject to:

$y_{i}\left( W^{T}x_{i}+b \right)\geq1-\xi_{i} (i=1,2,\ldots,n)$ (12)

where $w_{+}^{'}$, $w_{+}^{''}$, $w_{-}^{'}$, and $w_{-}^{''}$ are used as weights for training sets *P*, *LP*, *RN*, and *LP* respectively. The weight for *WN* equals one in equation 12. Let *Ψ* denote the weight vector as ($w_{+}^{'}$,$w_{+}^{''}$,$w_{-}^{'}$,$w_{-}^{''}$), we vary *c* and *Ψ* to obtain the empirical best parameter through 10 fold cross validation on whole disease gene set (3 fold cross validation on particular disease gene set), i.e. try different parameter values using the 9- fold training data and compute the classifiers’ performance using the remaining 1 fold validation set. The parameter values with the best average results will be set the final parameters values.

In particular, we vary *c* with $2^{-8}$, $2^{-7}$, $2^{-6}$, …,$2^{7}, 2^{8}$, and vary *Ψ* in following ways: *Ψ* is initialized by (1, 1, 1, 1), and then vary each *w* of *Ψ* by turns while keeping other *w* (*w*$\in$*Ψ*) stable. Firstly, we vary $w_{+}^{'}$ from $2^{-6}$, $2^{-5}$, $2^{-4}$, …,$2^{5}$, and we obtain the empirical optimal value $2^{b}$for $w_{+}^{'}$. Then, we vary $w_{+}^{''}$ with $2^{-6}$, $2^{-5}$, …,$2^{b}$, which guarantee the weight of *LP* is lower than that of *P*. After turning parameters $w_{+}^{'}$ and $w_{+}^{''}$ for positive weights, we vary $w_{-}^{'}$ and $w_{-}^{''}$ respectively following the same step as $w_{+}^{'}$ and $w_{+}^{''}$ in the range of $2^{-6}$, $2^{-5}$, …,$2^{b}$. We discover that it is good enough to tune $w_{-}^{'}$ and $w_{-}^{''}$ in this range from our multiple experimental trials. In summary, we set the values for $w_{+}^{'}$, $w_{+}^{''}$, $w_{-}^{'}$,$\mathrm{and} w_{-}^{''}$ in turn so that they can achieve best average performances using cross validation experiments.

We used the criteria of Weight SVM parameter tuning procedure in (Liu *et al*., 2011). In our experiments on general disease gene identification, we found that we could obtain the best performance when parameter *C* was around 256, $w_{+}^{'}$ from 1.1 to 1.9, $w_{+}^{''}$ and $w_{-}^{''}$ from 1 to 1.1, and $w_{-}^{'}$ from 1.1 to 1.2 (note we can run a number of times cross-validation to get the average values). For example, one best performance for general disease gene identification was achieved when *C* = 256, $w_{+}^{'}$=1.5, $w_{-}^{'}$=1.2, $w_{+}^{''}$=1 and $w_{-}^{''}$=1.1. We show the actual procedure for parameter tuning below:

1. Initialize ($w_{+}^{'}$*,*$w_{+}^{''}$*,*$w_{-}^{'}$*,*$w_{-}^{''}$) by (1,1,1,1);
2. Vary *C* with $2^{-8}$, $2^{-7}$, $2^{-6}$, …,$2^{7}, and 2^{8}$ to get best result using cross-validation.
3. Vary $w_{+}^{'}$ from $2^{-6}$, $2^{-5}$, $2^{-4}$, …,$2^{5}$ to obtain optimal value $2^{b}$for $w_{+}^{'}$;
4. Vary $w_{+}^{''}$ with $2^{-6}$, $2^{-5}$, …,$2^{b}$, to obtain value $2^{b^{'}}$, $b^{'}<b$;
5. Vary $w_{-}^{'}$ and $w_{-}^{''}$ respectively following the same step (3 and 4) as $w_{+}^{'}$ and $w_{+}^{''}$.

**Algorithm S.1** The procedure of parameter tuning in PUDI

1. **PUDI for disease genes prediction on additional disease groups**

Next, we further investigate the capability of PUDI to detect disease genes for specific disease classes. To ensure that there are sufficient positive training data, we have selected all the disease groups which have more than 20 disease genes (there are 8 such disease classes according to (Goh *et al.*, 2007)). We have listed the results of cardiovascular diseases and endocrine diseases in the paper. Here, we will show the results for the remaining 6 disease classes, namely, 1. Cancer diseases, 2. Metabolic diseases, 3. Neurological diseases, 4. Nutritional diseases, 5. Ophthalmological diseases, 6. Psychiatric diseases. The experimental results are listed in Table S.7.

**Table S.7 The performance comparison of six disease classes**

| **Diseases** | **Number** | **Method** | **F-measure** | **AUC** |
| --- | --- | --- | --- | --- |
| Cancer | 210 | PUDI | **72.4%** | **0.806** |
|  |  | ProDiGe | 69.5% | 0.708 |
|  |  | Smalter’s method | 66.6% | 0.778 |
|  |  | Xu’s method (1) | 63.7% | ~ |
| Metabolic | 263 | PUDI | **82.4%** | **0.897** |
|  |  | ProDiGe | 69.3% | 0.668 |
|  |  | Smalter’s method | 69.6% | 0.728 |
|  |  | Xu’s method (1) | 71.4% | ~ |
| Neurological | 217 | PUDI | **76.3%** | **0.843** |
|  |  | ProDiGe | 68.1% | 0.646 |
|  |  | Smalter’s method | 63.1% | 0.753 |
|  |  | Xu’s method (1) | 63.0% | ~ |
| Nutritional | 22 | PUDI | **72.7%** | 0.754 |
|  |  | ProDiGe | 66.4% | 0.695 |
|  |  | Smalter’s method | 69.4% | **0.769** |
|  |  | Xu’s method (1) | 65.6% | ~ |
| Ophthalmological | 163 | PUDI | **74.9%** | **0.842** |
|  |  | ProDiGe | 66.6% | 0.647 |
|  |  | Smalter’s method | 55.6% | 0.758 |
|  |  | Xu’s method (1) | 58.8% | ~ |
| Psychiatric | 26 | PUDI | **69.2%** | **0.751** |
|  |  | ProDiGe | 65.5% | 0.734 |
|  |  | Smalter’s method | 66.1% | 0.742 |
|  |  | Xu’s method (1) | 55.7% | ~ |

In Table S.7, the first column represents the diseases, the second column lists the number of confirmed disease genes existed, and the last three columns denote the different methods and corresponding performance in terms of F-measure and AUC. As we can see clearly from Table S.7, F-measure of our proposed PUDI is consistently better than ProDiGe, Smalter’s and Xu’s methods. To further analyze prediction performance of these methods, ROC curve plots are drawn in Figure S.1 and corresponding AUC from Table S.7, indicating that PUDI outperform ProDiGe, and Smalter’s method on most of eight disease groups. Since Xu’s method did not provide score measures for ranking genes for ROC curves, we were not able to compare this method with others.

**
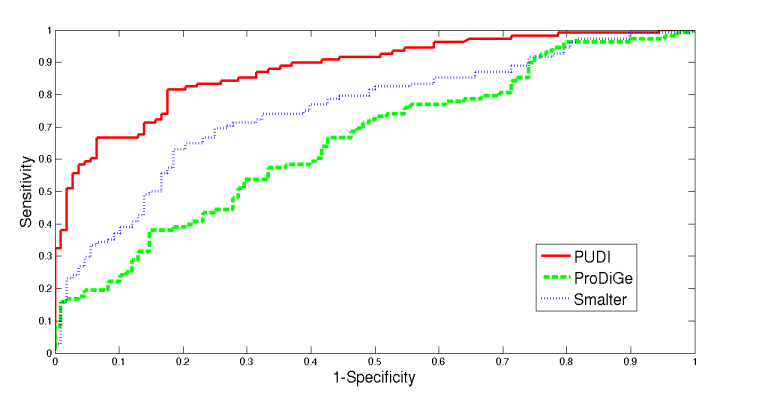
Figure S.1(a).** ROC curves on cancer disease category


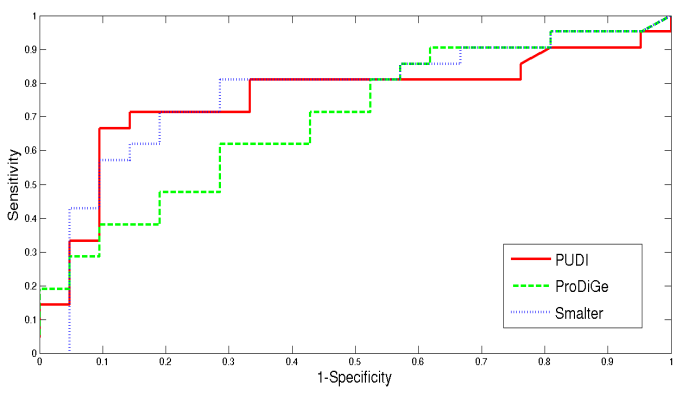


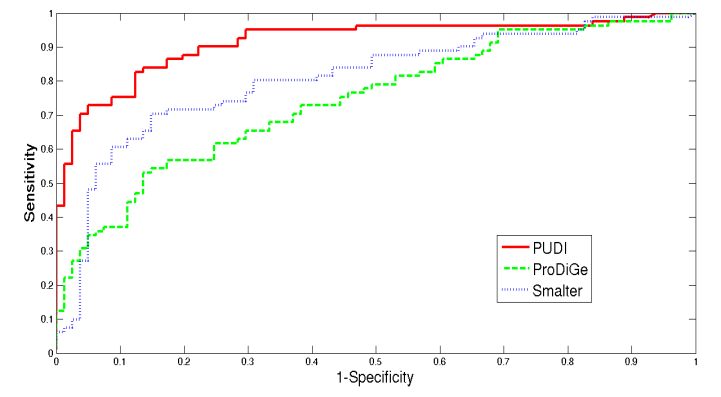


**Figure S.1(b).** ROC curves on cardiovascular disease category


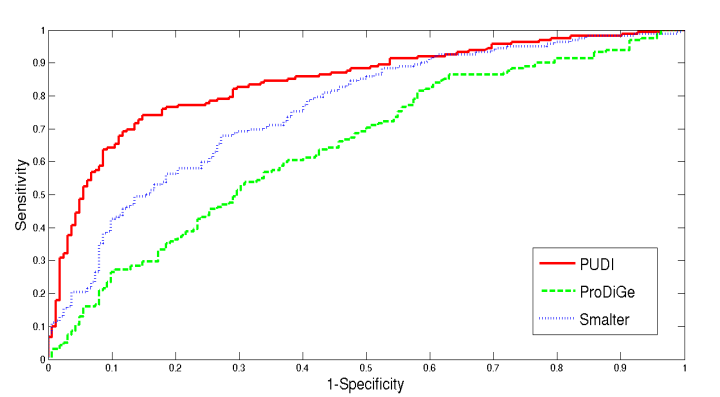

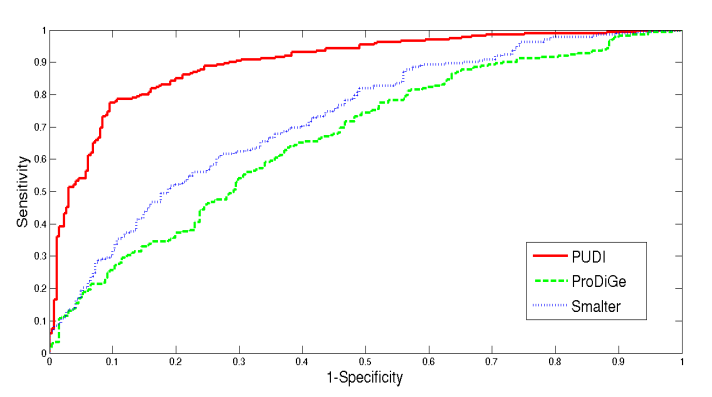


**
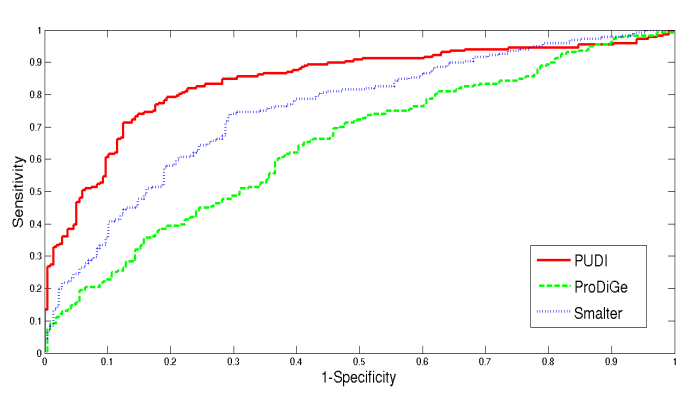

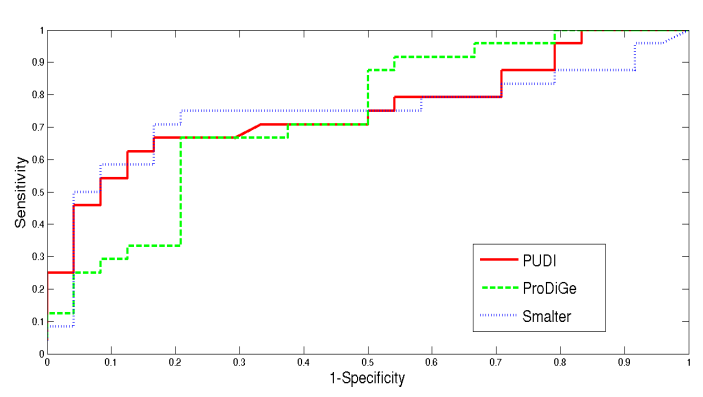
Figure S.1(c).** ROC curves on endocrine disease category

**Figure S.1(d).** ROC curves on metabolic disease category


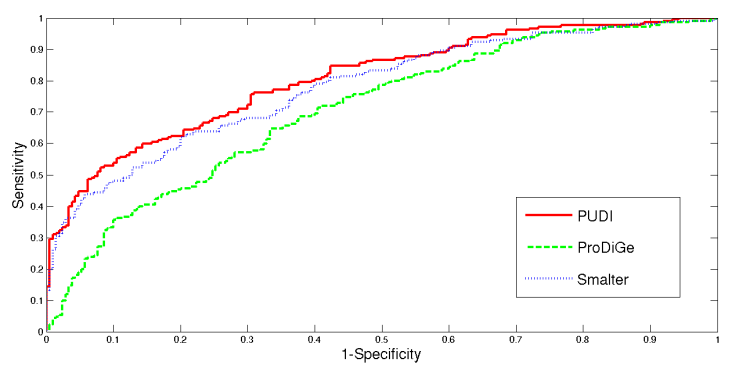
**Figure S.1(e).** ROC curves on neurological disease category

**Figure S.1(f).** ROC curves on nutritional disease category

**Figure S.1(g).** ROC curves on Ophthalmological disease category

**Figure S.1(h).** ROC curves on Psychiatric disease category

1. **The novel disease genes associated with endocrine disease**

We perform our proposed PUDI algorithm on endocrine disease and find that out of 11 predicted disease genes, three novel genes associated with endocrine diseases: EPHB6, CAMK2D, HEC6. Methylation-specific polymerase chain reaction (MSP) of EPHB6 is associated with breast cancer that is an endocrine-related cancer. In fact, studying the EPHB6 MSP is helpful for the prognosis and/or diagnosis of breast cancer (Fox *et al*., 2010). Calmodulin and calmodulin-dependent protein kinase II (CaMKII) plays important rules in neuroendocrine cell. In Lu *et al.* (Lu *et al*., 2011), CaMKII negatively contributes to the regulation of parathyroid hormone (PTH) secretion via a pathway. Finally, HEC6 has medical implication in metastatic neuroendocrine prostate cancer, breast cancer and metastatic colon carcinoma (Swearingen *et al*., 2003; Vias *et al*., 2008; Hartman *et al*., 2009).

1. **System schema of PUDI algorithm**


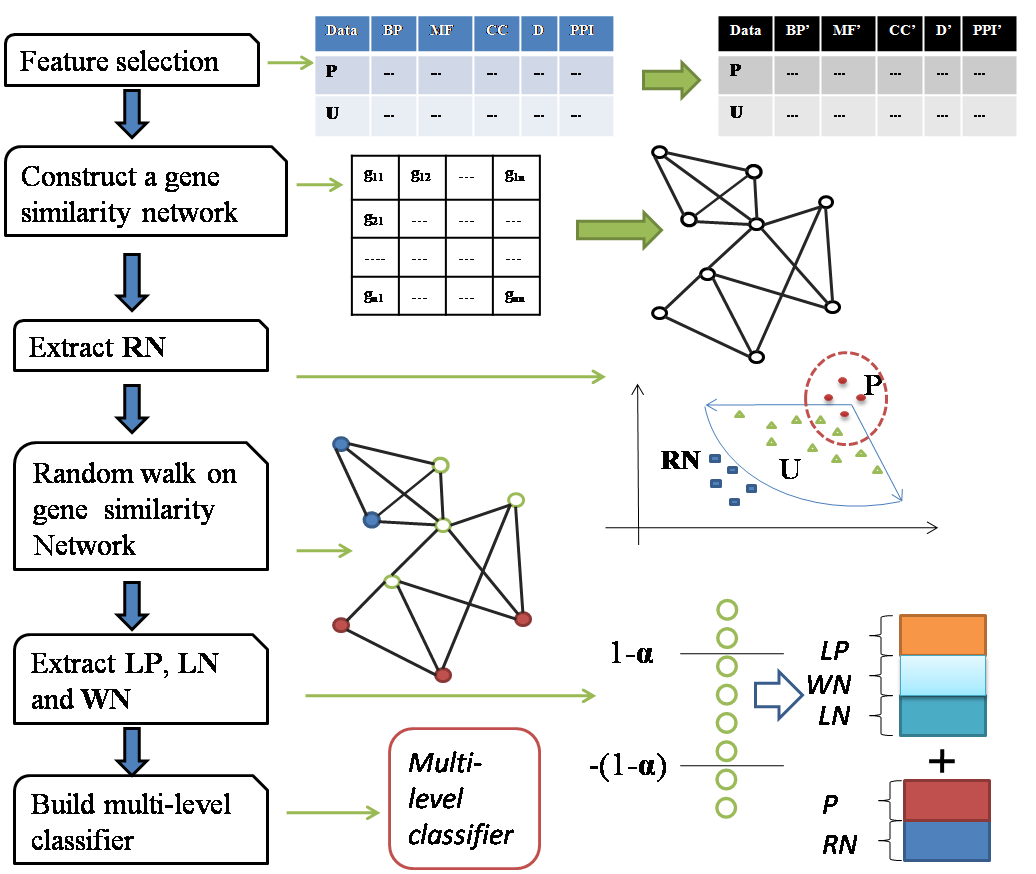
The system schema of PUDI algorithm is presented in Figure S.2. PUDI first apply a novel feature selection method to select useful features to distinguish gene samples in P from gene samples in U. Then reliable negative samples in RN, dissimilar with P in terms of various biological evidence (include protein domains (D), molecular functions (MF), biological processes (BP), cellular components (CC), as well as the genes’ topological properties in the protein interaction networks), are extracted from gene samples in U. After that, a gene similarity network is constructed based on the similarities between gene vectors. Random walk with restart algorithm is then run on the network for extracting likely positive set LP, likely negative set LN and weak negative set WN from samples in U according to their scores assigned by our algorithm. Finally a multi-level classifier is built for disease gene identification using P and four extracted sample set from U (RN, LP, LN, and WN) via weighted Support Vector Machine (WSVM).

**Figure S.2 The system schema of PUDI algorithm**

**The left panel consists of all the steps of PUDI algorithm. The right panel represents the results of data transformation**

The data flow of PUDI is shown in Figure S.3 to help readers understand the steps of PUDI.


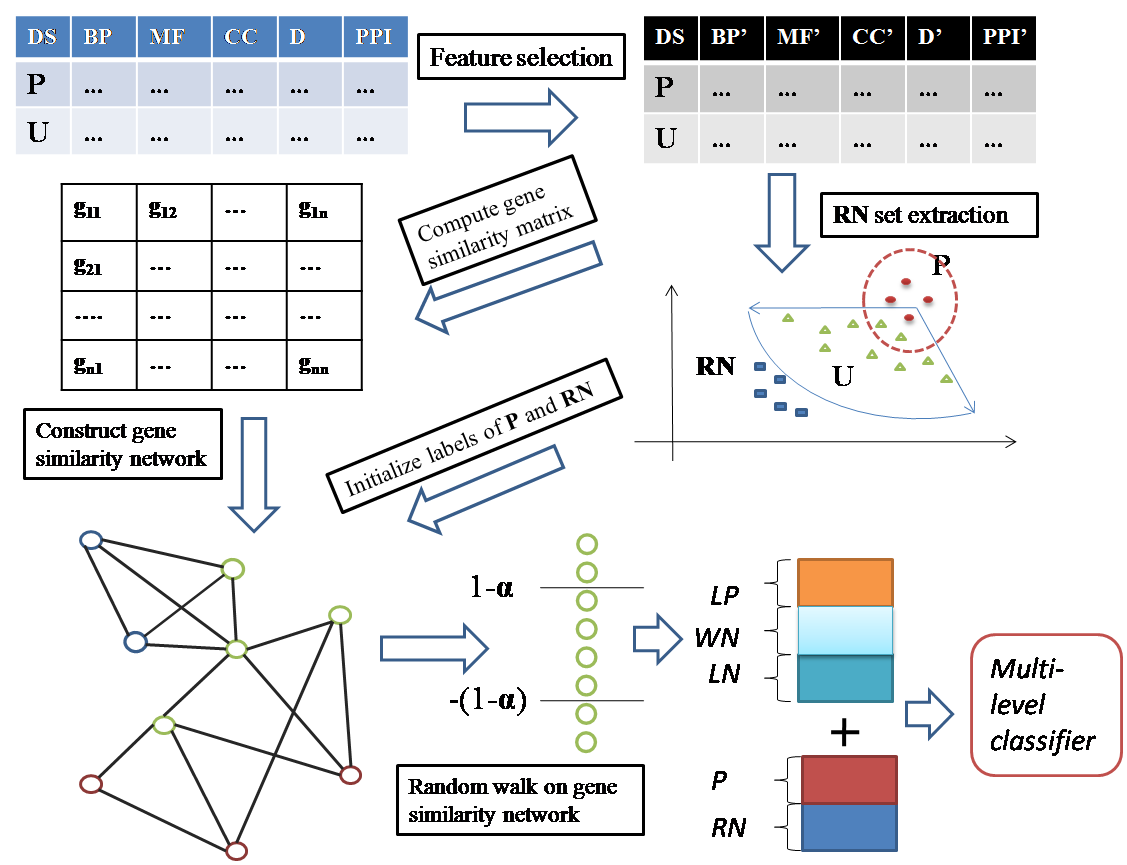


**Figure S.3** Data flow of PUDI algorithm

1. **The efficiency of different algorithms**

In this Section, we first discuss the time complexity of various computational methods for disease gene prediction and then show the actual time spent by each individual methods. We compare the time complexity of the new approach PUDI with three existing methods, namely ProDiGe, Smalter’s method and Xu’s method.

PUDI, ProDiGe and Smalter’s method are all SVM-based approaches and the training time complexity of SVM is O(*N^2^*) where *N* is number of training samples. For PUDI, it needs three additional steps: (i) to extract *RN* (with time complexity O(*N*)), (ii) to construct a gene similarity matrix and a gene similarity network (with time complexity O(*N^2^*)), and (iii) to run a random walk algorithm to extract *LN*, *LP* and *WN*. According to (Macropol, *et al*. 2009), Step (iii) has time complexity O(*w*N^2^*) in which *w* is number of iterations to converge. However, since *w* is typically very small (in our experiments *w*=20) compared to *N*, O(*w***N^2^*) can be reduced to O(*N^2^*). As such, the overall time complexity of PUDI is still O(*N^2^*). Similarly, the additional steps in ProDiGe and Smalter’s methods do not increase their time complexity as well, so they still end up with an overall time complexity of O(*N^2^*). Although Xu’s method is based on KNN algorithm, which classifies each target gene based on its similarities to all the other genes in the training set, the complexity of KNN algorithm is also O(*N^2^*). In summary, all the tools have exactly the same time complexity.

Next, we compare the actual running times using different tools across different disease groups. All the experiments were performed on the same machine with 1.83GHz CPU and 1GB (987MHz) memory.

**Table S.8** Speed comparisons using three algorithms

| **Disease Group** | **NO. of Samples** | **Approaches** | **Time (s)** |
| --- | --- | --- | --- |
| Cardiovascular disease dataset | 210 | PUDI | 15.410 |
|  | 210 | ProDiGe | 13.328 |
|  | 210 | Smalter’s method | 14.953 |
| Metabolic disease dataset | 526 | PUDI | 45.578 |
|  | 526 | ProDiGe | 34.000 |
|  | 526 | Smalter’s method | 41.015 |
| Neurological disease dataset | 434 | PUDI | 40.486 |
|  | 434 | ProDiGe | 32.538 |
|  | 434 | Smalter’s method | 34.75 |
| Ophthalmological disease dataset | 214 | PUDI | 17.316 |
|  | 214 | ProDiGe | 14.11 |
|  | 214 | Smalter’s method | 16.078 |
| Cancer disease dataset | 350 | PUDI | 29.075 |
|  | 350 | ProDiGe | 22.391 |
|  | 350 | Smalter’s method | 24.116 |

Table S.8 shows the actual running time for the three SVM-based methods. First, we observe that PUDI did spend 10%-20% more time than ProDiGe and Smalter’s method (see also Figure S.4), as it needed to perform a number of steps before building the multi-level SVM classifier. However, we also notice that the additional time spent was quite small, i.e. only a few more seconds for all the disease groups, and with that our PUDI was able to achieve at least 10%-20% improvement in terms of F-measure than the other existing methods on most of the specific disease gene groups. Furthermore, once the final classifiers are built, the efficiency of prediction procedures is more or less same among all these methods. As such, our PUDI method which can provide more accurate prediction is certainly preferable.

**Figure S.4** Running time using different algorithms

**References of Table 4**

1. Acar, K., Aksu, S., Beyazit, Y., Haznedaroglu, I., Koca, E., Cetiner, D., et al. (2008). Soluble platelet glycoprotein V in distinct disease states of pathological thrombopoiesis. *J Natl Med Assoc*., **100**(1):86-90.
2. Afonyushkin, T., Oskolkova, O., Philippova, M., Resink, T., Erne, P., Binder, B., et al. (2010). Oxidized Phospholipids Regulate Expression of ATF4 and VEGF in Endothelial Cells via NRF2-Dependent Mechanism: Novel Point of Convergence Between Electrophilic and Unfolded Protein Stress Pathways. *Arterioscler Thromb Vasc Biol*, **30**(5):1007-1013.
3. Attwell, S., Mills, J., Troussard, A., Wu, C., & Dedhar, S. (2003). Integration of cell attachment, cytoskeletal localization, and signaling by integrin-linked kinase (ILK), CH-ILKBP, and the tumor suppressor PTEN. *Mol Biol Cell*., **14**(12):4813-25.
4. Berger, G., Massé, J., & Cramer, E. (1996). Alpha-granule membrane mirrors the platelet plasma membrane and contains the glycoproteins Ib, IX, and V. *Blood*, **87**(4):1385-95.
5. Bertrand, F., Vogtenhuber, C., Shah, N., & LeBien, T. ( 2001). Pro-B-cell to pre-B-cell development in B-lineage acute lymphoblastic leukemia expressing the MLL/AF4 fusion protein. *Blood* , **98**(12):3398-405.
6. Bradwejn, J., Koszycki, D., & Meterissian, G. (1990). Cholecystokinin-tetrapeptide induces panic attacks in patients with panic disorder. *Can J Psychiatry*., **35**(1):83-5.
7. Bradwejn, J., Koszycki, D., Couëtoux du Tertre, A., Paradis, M., & Bourin, M. (1994). Effects of flumazenil on cholecystokinin-tetrapeptide-induced panic symptoms in healthy volunteers. *Psychopharmacology (Berl)* , **114**(2):257-6.
8. Brancaccio, M., Menini, N., Bongioanni, D., Ferretti, R., De Acetis, M., Silengo, L., et al. (2003 ). Chp-1 and melusin, two CHORD containing proteins in vertebrates. *FEBS Lett*. , **551**(1-3):47-52.
9. Chen, C., Hilden, J., Frestedt, J., Domer, P., Moore, R., Korsmeyer, S., et al. (1993). The chromosome 4q21 gene (AF-4/FEL) is widely expressed in normal tissues and shows breakpoint diversity in t(4;11)(q21;q23) acute leukemia. *Blood*, **82**(4):1080-5.
10. Chen, Q. B. (2006). Increased WSB1 copy number correlates with its over-expression which associates with increased survival in neuroblastoma. *Genes Chromosomes Cancer*, **45**(9):856-62.
11. Chen, Z., & Wang, H. ( 2004). Alteration of cytokeratin 7 and cytokeratin 20 expression profile is uniquely associated with tumorigenesis of primary adenocarcinoma of the small intestine. *Am J Surg Pathol*., **28**(10):1352-9.
12. Cheuk, W., Kwan, M., Suster, S., & Chan, J. (2001). Immunostaining for thyroid transcription factor 1 and cytokeratin 20 aids the distinction of small cell carcinoma from Merkel cell carcinoma, but not pulmonary from extrapulmonary small cell carcinomas. *Arch Pathol Lab Med*., **125**(2):228-31.
13. Cuenda, A., Cohen, P., Buée-Scherrer, V., & Goedert, M. (1997). Activation of stress-activated protein kinase-3 (SAPK3) by cytokines and cellular stresses is mediated via SAPKK3 (MKK6); comparison of the specificities of SAPK3 and SAPK2 (RK/p38). *EMBO J*., **16**(2):295-30.
14. Eser, D., di Michele, F., Zwanzger, P., Pasini, A., Baghai, T., Schüle, C., et al. (2005). Panic induction with cholecystokinin-tetrapeptide (CCK-4) Increases plasma concentrations of the neuroactive steroid 3alpha, 5alpha tetrahydrodeoxycorticosterone (3alpha, 5alpha-THDOC) in healthy volunteers. *Neuropsychopharmacology*, **30**(1):192-5.
15. Fox, BP. & Kanpal RP. (2010) DNA-Based Assay for EPHB6 Expression in Breast Carcinoma Cells as a Potential Diagnostic Test for Detecting Tumor Cells in Circulation. Cancer Genomics and Proteomics, **7**: 9-16.
16. Gil, J., & Esteban, M. (2000). Induction of apoptosis by the dsRNA-dependent protein kinase (PKR): mechanism of action. *Apoptosis*. , **5**(2):107-14.
17. Goh, K. *et al*. (2007). The human disease network. *PNAS*, **104**(21): 8685-8690.d
18. Hamann, J., Wishaupt, J., van Lier, R., Smeets, T., Breedveld, F., & Tak, P. (1999). Expression of the activation antigen CD97 and its ligand CD55 in rheumatoid synovial tissue. *Arthritis Rheum*. , **42**(4):650-8.
19. Hiasa, Y., . Kamegaya, Y., Nuriya, H., Onji, M., Kohara, M., Schmidt, E., et al. (2003). Protein kinase R is increased and is functional in hepatitis C virus-related hepatocellular carcinoma. *Am J Gastroenterol*. , **98**(11):2528-34.
20. Hoang-Vu, C., Bull, K., Schwarz, I., Krause, G., Schmutzler, C., Aust, G., et al. (1999). Regulation of CD97 protein in thyroid carcinoma. *J Clin Endocrinol Metab*. , **84**(3):1104-9.
21. Ji, H., Isacson, C., Seidman, J., Kurman, R., & Ronnett, B. (2002). Cytokeratins 7 and 20, Dpc4, and MUC5AC in the distinction of metastatic mucinous carcinomas in the ovary from primary ovarian mucinous tumors: Dpc4 assists in identifying metastatic pancreatic carcinomas. *Int J Gynecol Pathol*. , **21**(4):391-400.
22. Koszycki, D., Zacharko, R., & Bradwejn, J. (1996). Influence of personality on behavioral response to cholecystokinin-tetrapeptide in patients with panic disorder. *Psychiatry Res*., **62**(2):131-8.
23. Le Mellédo, J., Merani, S., Koszycki, D., Bellavance, F., Palmour, R., Gutkowska, J., et al. (1999). Sensitivity to CCK-4 in women with and without premenstrual dysphoric disorder (PMDD) during their follicular and luteal phases. *Neuropsychopharmacology* , **20**(1):81-91.
24. Lee, S., Bablanian, R., & Esteban, M. (1996). Regulated expression of the interferon-induced protein kinase p68 (PKR) by vaccinia virus recombinants inhibits the replication of vesicular stomatitis virus but not that of poliovirus. *J Interferon Cytokine Res.*, **16**(12):1073-8.
25. Li, Q., Frestedt, J., & Kersey, J. (1998). AF4 encodes a ubiquitous protein that in both native and MLL-AF4 fusion types localizes to subnuclear compartments. *Blood*, **92**(10):3841-7.
26. Libermann TA., Zerbini LF. (2006). Targeting transcription factors for cancer gene therapy. *Curr Gene Ther* **6**(1): 17–33.
27. Lu, M., et al. (2011) Calmodulin and calmodulin-dependent protein kinase II inhibit hormone secretion in human parathyroid adenoma. *J Endocrinol*, **208**: 31-39
28. Mayer, J., & Beardsley, D. (1996). Varicella-associated thrombocytopenia: autoantibodies against platelet surface glycoprotein V. *Pediatr Res*., **40**(4):615-9.
29. Min, J., Li, S., Sen, G., & Krug, R. (2007). A site on the influenza A virus NS1 protein mediates both inhibition of PKR activation and temporal regulation of viral RNA synthesis. *Virology*., **363**(1):236-43.
30. Modderman, P., Admiraal, L., Sonnenberg, A., & von dem Borne, A. (1992). Glycoproteins V and Ib-IX form a noncovalent complex in the platelet membrane. *J Biol Chem*., **267**(1):364-9.
31. Mossie, K., Jallal, B., Alves, F., Sures, I., Plowman, G., & Ullrich, A. (1995). Colon carcinoma kinase-4 defines a new subclass of the receptor tyrosine kinase family. *Oncogene* , **11**(10):2179-8.
32. Nagai, M., Fregnani, J., Netto, M., Brentani, M., & Soares, F. (2007). Down-regulation of PHLDA1 gene expression is associated with breast cancer progression. *Breast Cancer Res Treat.* , **106**(1):49-56.
33. Nakamura, T., Alder, H., Gu, Y., & Prasad, R. e. (1993). Genes on chromosomes 4, 9, and 19 involved in 11q23 abnormalities in acute leukemia share sequence homology and/or common motifs. *Proc Natl Acad Sci U S A* , **90**(10):4631-5.
34. Palumbo, V., Segat, L., Padovan, L., & al., e. (2009). Melusin gene (ITGB1BP2) nucleotide variations study in hypertensive and cardiopathic patients. *BMC Med Genet.* , **10**:140.
35. Peschon, J., Behringer, R., Cate, R., Harwood, K., Idzerda, R., Brinster, R., et al. (1992). Directed expression of an oncogene to Sertoli cells in transgenic mice using mullerian inhibiting substance regulatory sequences. *Mol Endocrinol* , **6**(9):1403-11.
36. Bradwejn, J.; Koszycki, D.; Annable, L.; Couëtoux du Tertre, A.; Reines, S.; Karkanias, C. (1992). A dose-ranging study of the behavioral and cardiovascular effects of CCK-tetrapeptide in panic disorder. *Biol Psychiatry*, **32**(10):903-12.
37. Rakeman, A., & Anderson, K. (2006). Axis specification and morphogenesis in the mouse embryo require Nap1, a regulator of WAVE-mediated actin branching. *Development*, **133**(16):3075-3083.
38. Ravanat, C., Morales, M., Azorsa, D., Moog, S., Schuhler, S., Grunert, P., et al. (1997). Gene cloning of rat and mouse platelet glycoprotein V: identification of megakaryocyte-specific promoters and demonstration of functional thrombin cleavage. *Blood*. , **89**(9):3253-62.
39. Ross, M., Zhou, X., Song, G., & al., e. (2003). Classification of pediatric acute lymphoblastic leukemia by gene expression profiling. *Blood*. , **102**(8):2951-9.
40. Roth, G., Church, T., McMullen, B., & Williams, S. (1990). Human platelet glycoprotein V: a surface leucine-rich glycoprotein related to adhesion. *Biochem Biophys Res Commun* , **170**(1):153-61.
41. Scheuer K., *et al*. (1996). "Cortical NMDA Receptor Properties and Membrane Fluidity Are Altered in Alzheimer's Disease". Dementia **7**(4): 210–214.
42. Sepulveda, J., & Wu, C. (2006). The parvins. *Cell Mol Life Sci*. , **63**(1):25-35.
43. Shi, Q., Wilcox, D., Morateck, P., Fahs, S., Kenny, D., & Montgomery, R. (2004). Targeting platelet GPIbalpha transgene expression to human megakaryocytes and forming a complete complex with endogenous GPIbbeta and GPIX. *J Thromb Haemost* , **2**(11):1989-97.
44. Smagur, A., Szary, J., & Szala, S. (2005). Recombinant angioarrestin secreted from mouse melanoma cells inhibits growth of primary tumours. *Acta Biochim Pol*., **52**(4):875-9.
45. Smith, K., Mezhir, J., Bickenbach, K., & al., e. (2006). Activated MEK suppresses activation of PKR and enables efficient replication and in vivo oncolysis by Deltagamma(1)34.5 mutants of herpes simplex virus 1. *J Virol*. , **80**(3):1110-20.
46. Steinert, M., Wobus, M., Boltze, C., Schütz, A., Wahlbuhl, M., Hamann, J., et al. (2002). Expression and regulation of CD97 in colorectal carcinoma cell lines and tumor tissues. *Am J Pathol*. , **161**(5):1657-67.
47. Steinert, M., Wobus, M., Boltze, C., Schütz, A., Wahlbuhl, M., Hamann, J., et al. (2002). Expression and regulation of CD97 in colorectal carcinoma cell lines and tumor tissues. *Am J Pathol*. , **161**(5):1657-6.
48. Takahashi, M.; Takahashi, Y.; Takahashi, K., et al. ( 2007). CXCL14 enhances insulin-dependent glucose uptake in adipocytes and is related to high-fat diet-induced obesity. *Biochem Biophys Res Commun*, **364**(4):1037-1042.
49. Vajda, N., Brimacombe, K., LeMasters, K., & Ladd, A. (2009). Muscleblind-like 1 is a negative regulator of TGF-β-dependent epithelial–mesenchymal transition of atrioventricular canal endocardial cells. *Developmental Dynamics*, **238**(12): 3266-3272.
50. van Megen, H., Westenberg, H., den Boer, J., Slaap, B., & Scheepmakers, A. (1997). Effect of the selective serotonin reuptake inhibitor fluvoxamine on CCK-4 induced panic attacks. *Psychopharmacology* *(Berl)*, **129**(4):357-64.
51. [Wu, G.](http://en.wikipedia.org/w/index.php?title=Ge_Wu&action=edit&redlink=1) (2010). [Assays with GPCRs.](http://books.google.com/books?id=qxKqC1aGLBIC&pg=PA265) Assay Development: Fundamentals and Practices. *John Wiley and Sons*. pp. 265–285
52. Xu, Y., Liu, Y., & Yu, Q. ( 2004). Angiopoietin-3 inhibits pulmonary metastasis by inhibiting tumor angiogenesis. *Cancer* *Res*., **64**(17):6119-26.
53. Yamamoto, S., Zaitsu, M., Ishii, E., Yatsuki, H., Mizutani, S., Eguchi, M., et al. (1998). High frequency of fusion transcripts of exon 11 and exon 4/5 in AF-4 gene is observed in cord blood, as well as leukemic cells from infant leukemia patients with t(4;11)(q21;q23). *Leukemia*, **12**(9):1398-403.
